# Supplementary material for: Complex Interplay between Epitope Specificity and Isotype Dictates the Biological Activity of Anti-human CD40 Antibodies
Source: Cancer Cell. 2018 Apr 9;33(4):664–675.e4. doi: 10.1016/j.ccell.2018.02.009 (PMC5896247; doi:10.1016/j.ccell.2018.02.009)
Supplement: Document S1. Figures S1–S3 and Table S1 [file mmc1.pdf]

Cancer Cell, Volume *33*

Supplemental Information

Complex Interplay between Epitope Specificity and Isotype Dictates the Biological Activity of Anti-human CD40 Antibodies

Xiaojie Yu, H.T. Claude Chan, Christian M. Orr, Osman Dadas, Steven G. Booth, Lekh N. Dahal, Christine A. Penfold, Lyn O'Brien, C. Ian Mockridge, Ruth R. French, Patrick Duriez, Leon R. Douglas, Arwen R. Pearson, Mark S. Cragg, Ivo Tews, Martin J. Glennie, and Ann L. White

Table S1. Related to Figure 2.

	ChiLob 7/4 Fab:CD40 Complex
**Data collection**	
**Space group**	P 3_1 2 1
**Cell dimensions**	
**a, b, c (Å)**	158.75 158.75 96.62
**a, b, g (°)**	90 90 120
**Resolution (Å)**	51.96 - 3.00 (3.08-3.00)
**R_{sym} or R_{merge}**	0.157 (1.445)
**I / sI**	13.0 (32.9)
**Completeness (%)**	100 (98.4)
**Redundancy**	13.7 (11.4)
**Refinement**	
**Resolution (Å)**	3.00
**No. reflections**	27540
**R_{work} / R_{free}**	0.190 / 0.237
**No. atoms**	
**Protein**	4091
**Ligand/ion**	0
**Water**	23
**B-factors**	
**Protein**	87.03
**Ligand/ion**	0
**Water**	78.14
**R.m.s. deviations**	
**Bond lengths (Å)**	0.0169
**Bond angles (°)**	2.135

Data Collection and Refinement Statistics of ChiLob 7/4 Fab':CD40 Complex (PDB ID 6FAX). High resolution shell statistics shown in parenthesis. 5% of reflections were used for calculation of Rfree.

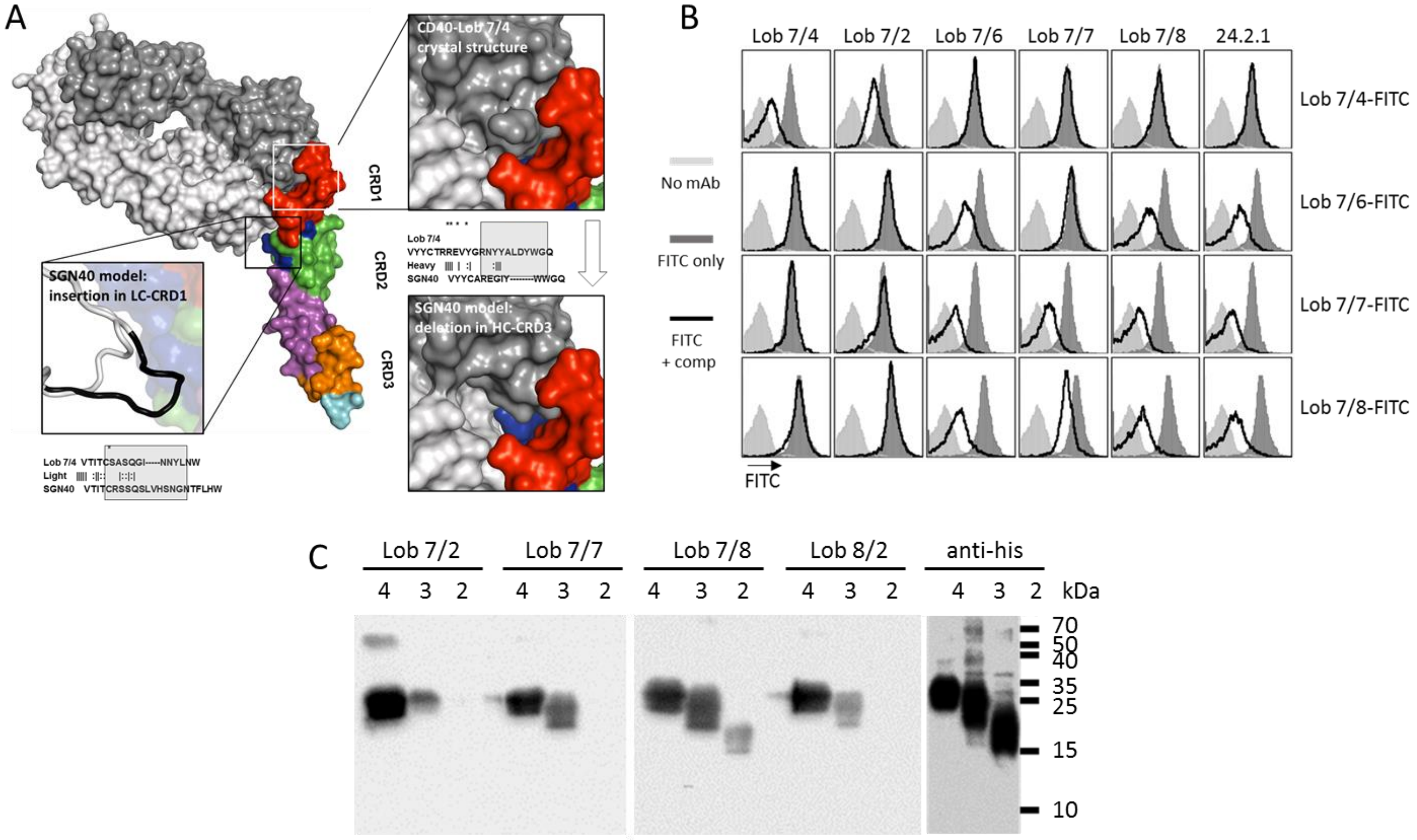

Figure S1. Epitope mapping of hCD40 mAb. Related to Figure 3. (A) Modelling of SGN40 binding onto the ChiLob 7/4:CD40 crystal structure. Surface view of the structure of the CD40:ChiLob 7/4 complex with CRD regions of CD40 coloured and mapped as in Figure 2 with various insets highlighting key parts of the interaction. Below are models produced to show the predicted structural differences apparent in the SGN40 and ChiLob 7/4 Fab' based on loop length changes in the heavy chain CRD3 and the light chain CRD1. Panels: Top right panel highlights the binding of ChiLob 7/4 to CD40 in the crystal structure. Note: The LC CDR3 of ChiLob 7/4 binds into a pocket on the CRD1-B2 of CD40 and the light chain CRD1 of ChiLob 7/4 binds in a pocket of CD40 CDR1-A1. Below is shown the sequence alignment between the HC CDR3 of ChiLob 7/4 and SGN40 with the key binding residues in the structure of ChiLob 7/4 starred. Below (bottom right) shows how the 8 amino-acid deletion in the SGN40 HC would prevent it making the same interaction with the pocket of CRD1-B2 of

CD40 as ChiLob 7/4. The bottom left panel shows a modelled representation of how the 5 amino-acid insertion of the SGN40 LC CDR1 (bottom loop, black) would impair binding in this region. Below is the sequence alignment between the LC CDR1 of ChiLob 7/4 and SGN40 with the key binding residue starred. (B) hCD40Tg mouse B cells were incubated with FITC-labelled Lob 7/4, Lob 7/6, Lob 7/7 or Lob 7/8 as indicated in the absence or presence of a 10-fold excess of competitor (comp) mAb indicated above each plot. Cell labelling was assessed by flow cytometry; light grey, unlabelled cells; dark grey, FITC-labelled mAb alone; black line, FITC-labelled mAb + competitor. (C) C-terminally His-tagged CD40 proteins consisting of 4, 3, or 2 CRD domains (4 = full length; 3 = CRD1 deleted; 2 = CRD1 and 2 deleted) were analysed by Western blotting with the mAb indicated above each plot used for detection. A composite image from multiple blots is shown.

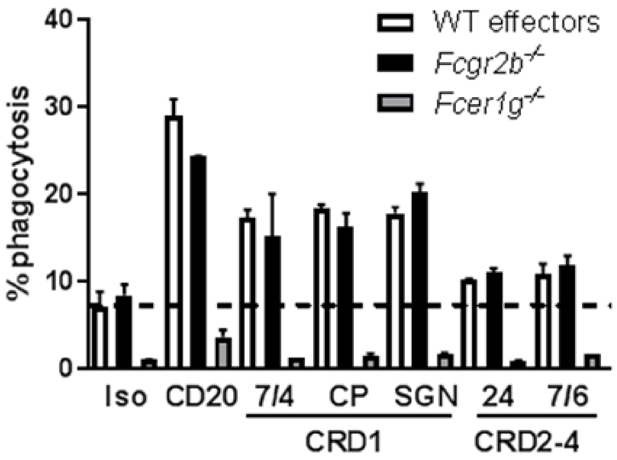

Figure S2. anti-hCD40 mAb-mediated phagocytosis. Related to Figure 4. Purified hCD40Tg mouse splenic B cells were opsonised with the indicated hCD40 mAb, mouse CD20 mAb, or isotype control, all m2a isotype, before incubation with bone marrow derived mouse macrophages from WT, *Fcgr2b*$^{-/-}$ or *Fcer1g*$^{-/-}$ mice. Phagocytosis was measured by flow cytometry as described in STAR Methods. Results (mean ± SEM) from one of 2 independent experiments are shown, * $p<0.05$ versus isotype control for WT cells.

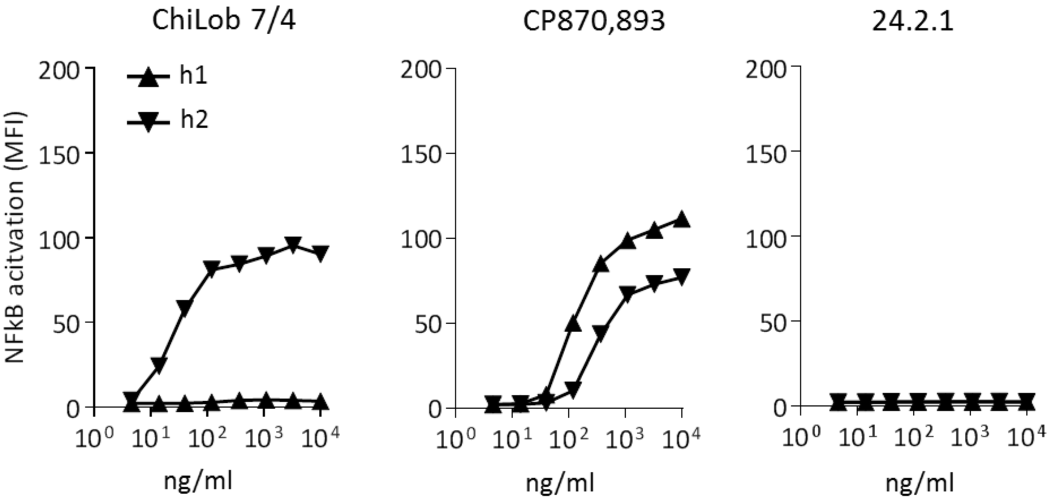

Figure S3. NF- κ B activation by anti-hCD40 mAb. Related to Figure 5. NF-κB/Jurkat/GFP reporter cells stably transfected with hCD40 were incubated with increasing concentrations of the indicated anti-hCD40 mAb of h1 or h2 isotypes for 8 hours, and then the level of NF- κ B activation was assessed by GFP quantification using flow cytometry. Mean ± SEM of triplicates, data representative of at least 2 independent experiments.
