# Supplementary material for: Complex Interplay between Epitope Specificity and Isotype Dictates the Biological Activity of Anti-human CD40 Antibodies
Source: Cancer Cell. 2018 Apr 9;33(4):664–675.e4. doi: 10.1016/j.ccell.2018.02.009 (PMC5896247; doi:10.1016/j.ccell.2018.02.009)
Supplement: Document S2. Article plus Supplemental Information [file mmc2.pdf]

Cancer Cell

Article

Complex Interplay between Epitope Specificity and Isotype Dictates the Biological Activity of Anti-human CD40 Antibodies

Graphical Abstract

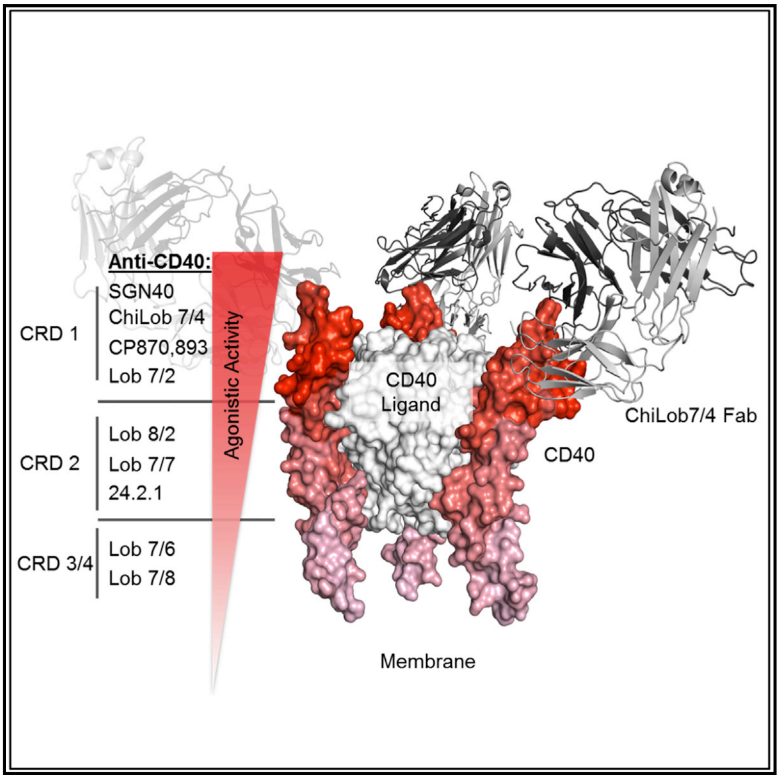

Highlights

- Epitope in combination with isotype governs hCD40 mAb activity
- CRD1 binding mAbs are agonistic as IgG2 or with FcγRIIB crosslinking
- CRD2-4 binding mAbs display antagonistic properties
- Structure of receptor complex provides insights into agonistic function

Authors

Xiaojie Yu, H.T. Claude Chan, Christian M. Orr, ..., Ivo Tews, Martin J. Glennie, Ann L. White

Correspondence

ann.white@ucb.com

In Brief

CD40 agonist mAbs are being investigated for cancer treatment, whereas antagonistic mAbs are under investigation for the treatment of autoimmune and inflammatory conditions. Yu et al. show that the activity of a CD40 mAb is determined by an interplay between the location of its epitope within CD40 and its isotype.

Yu et al., 2018, Cancer Cell 33, 664–675
April 9, 2018 © 2018 The Authors. Published by Elsevier Inc.
https://doi.org/10.1016/j.ccell.2018.02.009

CellPress

Cancer Cell
Article

Complex Interplay between Epitope Specificity and Isotype Dictates the Biological Activity of Anti-human CD40 Antibodies

Xiaojie Yu,[1] H.T. Claude Chan,[1] Christian M. Orr,[1] Osman Dadas,[1] Steven G. Booth,[1] Lekh N. Dahal,[1] Christine A. Penfold,[1] Lyn O'Brien,[1,7] C. Ian Mockridge,[1] Ruth R. French,[1] Patrick Duriez,[2] Leon R. Douglas,[2] Arwen R. Pearson,[3] Mark S. Cragg,[1,4] Ivo Tews,[4,5] Martin J. Glennie,[1,8] and Ann L. White[1,6,8,9,*]

[1]Antibody and Vaccine Group, Cancer Sciences Unit, University of Southampton Faculty of Medicine, Southampton SO16 6YD, UK
[2]Protein Core Facility, University of Southampton Faculty of Medicine, Southampton SO16 6YD, UK
[3]Hamburg Centre for Ultrafast Imaging & Institute for Nanostructure and Solid State Physics, University of Hamburg, 20146 Hamburg, Germany
[4]Institute for Life Sciences, University of Southampton, Southampton SO17 1BJ, UK
[5]Biological Sciences, University of Southampton, Southampton SO17 1BJ, UK
[6]Present address: UCB-Celltech, 216 Bath Road, Slough, SL1 3WE, UK
[7]Present address: Dstl, Porton Down, Salisbury, SP4 0JQ, UK
[8]Senior author
[9]Lead Contact
*Correspondence: ann.white@ucb.com
https://doi.org/10.1016/j.ccell.2018.02.009

SUMMARY

Anti-CD40 monoclonal antibodies (mAbs) that promote or inhibit receptor function hold promise as therapeutics for cancer and autoimmunity. Rules governing their diverse range of functions, however, are lacking. Here we determined characteristics of nine hCD40 mAbs engaging epitopes throughout the CD40 extracellular region expressed as varying isotypes. All mAb formats were strong agonists when hyper-crosslinked; however, only those binding the membrane-distal cysteine-rich domain 1 (CRD1) retained agonistic activity with physiological Fc gamma receptor crosslinking or as human immunoglobulin G2 isotype; agonistic activity decreased as epitopes drew closer to the membrane. In addition, all CRD2-4 binding mAbs blocked CD40 ligand interaction and were potent antagonists. Thus, the membrane distal CRD1 provides a region of choice for selecting CD40 agonists while CRD2-4 provides antagonistic epitopes.

INTRODUCTION

Monoclonal antibodies (mAbs) that target regulatory receptors to modulate immune responses are revolutionizing the treatment of cancer and autoimmune disease (Chan and Carter, 2010; Schaer et al., 2014; Sharma and Allison, 2015). One promising target is CD40, a member of the tumor necrosis factor receptor superfamily (TNFRSF) expressed on cells including B cells and specialized antigen-presenting cells (Grewal and Flavell, 1998). Agonistic mAbs that stimulate CD40 signaling are in development as cancer therapeutics designed to potentiate anti-tumor immunity (Remer et al., 2017; Vonderheide and Glennie, 2013), with the most encouraging results coming with CP870,893 in pancreatic cancer (Beatty et al., 2011). Conversely, antagonistic mAbs that inhibit CD40 function are under investigation for the treatment of autoimmune and inflammatory conditions (Croft et al., 2013). The rules that govern whether a particular mAb will possess agonistic or antagonistic properties, however, are currently unclear.

The ligand for CD40 (CD40L/CD154) is trimeric and believed to initiate CD40 activation by clustering the receptor in the cell membrane allowing recruitment of TNFR-associated factors

Significance

Monoclonal antibodies (mAbs) that regulate immune responses are providing potent therapeutics for treating cancer and autoimmune disease. One target in development is CD40, a tumor necrosis factor receptor (TNFR) superfamily member expressed on antigen-presenting cells involved in regulating adaptive immunity. mAbs targeting this receptor show diverse activities, from strong agonism to powerful antagonism; however, the rules determining activity are unclear. Here we demonstrate that a complex interplay between the location of the mAb epitope within CD40 and the isotype determines these differing levels of activity. Such detailed understanding of mAb mechanism of action will help guide the development of the next generation of immuno-therapeutics targeting CD40 and potentially other members of the TNFR superfamily.

 © 2018 The Authors. Published by Elsevier Inc.
This is an open access article under the CC BY license (http://creativecommons.org/licenses/by/4.0/).

leading to immune activation (Werneburg et al., 2001). We have investigated the pathways by which agonistic anti-human (h) CD40 mAb can mimic this process and have demonstrated two distinct mechanisms, both influenced by the mAb constant region (White et al., 2011, 2015). The first is mediated by hyper-crosslinking of the mAb Fc through interaction with Fc gamma receptors (FcγR) (White et al., 2011). In *in vivo* mouse models crosslinking is mediated by the inhibitory FcγR, FcγRIIB. The anti-mouse (m) CD40 mAbs 3/23 (White et al., 2011) and IC10 (Li and Ravetch, 2011) are agonistic when expressed as isotypes that engage mouse FcγRIIB with relatively high affinity (mouse IgG1 [immunoglobulin G1] [m1] or rat IgG2a [r2a]), but not as isotypes with low FcγRIIB affinity (m2a or human IgG1 [h1]) or in the absence of FcγRIIB expression (Li and Ravetch, 2011; White et al., 2011). FcγRIIB is thought to act as a hyper-crosslinking scaffold for the mAbs to enhance clustering of CD40 in the membrane (Beers et al., 2016; White et al., 2013). Its predominant role *in vivo* likely reflects its bioavailability as other FcγR can mediate this function *in vitro*, at least when expressed at sufficient surface density (White et al., 2011; Wilson et al., 2011).

The second mechanism through which hCD40 mAbs can elicit activity is independent of FcγR, but dependent upon the hinge region of human IgG2 (h2) (White et al., 2015). h2 can adopt alternative disulfide bonding arrangements in its hinge that exhibit differences in flexibility and conformation (Dillon et al., 2008; Martinez et al., 2008; Wypych et al., 2008). We previously demonstrated that the hCD40 mAb ChiLob 7/4 becomes highly agonistic in the absence of FcγR-mediated hyper-crosslinking when "locked" in an h2 "B" conformation, where the two F(ab) arms are disulfide-linked to the hinge (Allen et al., 2009; White et al., 2015). h2B can agonize the receptor *in vivo* when administered as a F(ab')$_2$ fragment, potently stimulating antigen-specific CD8 T cell proliferation in wild-type (WT) or *Fcgr2b*$^{-/-}$ (knockout [KO]) mice, unequivocally demonstrating its FcγR-independent activity. Similarly, F(ab')$_2$ fragments of the clinical h2 hCD40 mAb CP870,893 also retain agonistic activity (Richman and Vonderheide, 2014), confirming that FcγR-mediated hyper-crosslinking is not obligatory for hCD40 mAb activity. We hypothesize that the compact h2B conformation promotes close packaging of CD40 molecules in the membrane allowing efficient TRAF recruitment and receptor activation even when FcγR are absent (Beers et al., 2016; White et al., 2015).

These isotype-dependent rules of agonistic activity have been shown for multiple mAb targeting CD40 and other TNFR (Li and Ravetch, 2011, 2012; White et al., 2015; Wilson et al., 2011; Xu et al., 2003), suggesting a common mechanism of action. However, they were established by dissecting modes of activity of known agonists. It is not clear whether all mAbs directed against CD40 or other TNFRSF members will be agonistic as m1 or h2 isotypes, or whether additional factors dictate activity. In particular, although epitope specificity clearly plays an important role in anti-hCD40 mAb function (Barr and Heath, 2001; Bjorck et al., 1994; Ellmark et al., 2002; Malmborg Hager et al., 2003; Yamniuk et al., 2016), the interaction between isotype and epitope in dictating anti-TNFR mAb activity has not been systematically investigated. Likewise, the rules governing antagonistic anti-hCD40 mAb behavior are not clear. True antagonism likely necessitates the ability to prevent CD40L-mediated receptor activation, suggesting that epitope specificity and the ability to prevent CD40L binding are inter-related.

The goal of this study was to define the relationship between epitope location and isotype in dictating the biological activity of hCD40 mAbs.

RESULTS

Epitope and Agonist Activity

To examine the role of epitope specificity in the agonistic activity of hCD40 mAbs, we assembled a panel of nine mAbs expressed with the m1 constant domain known to confer agonistic activity through FcγRIIB engagement (Li and Ravetch, 2011; White et al., 2011). We then compared their ability to stimulate B cell proliferation *in vitro* and CD8 T cell expansion *in vivo*. Three of these mAbs, Lob 7/4-m1, SGN40-m1, and CP870,893-m1 were derived from ChiLob 7/4, SGN40 (dacetuzumab), and CP870,893, respectively, all of which have been used in clinical trials (Hussein et al., 2010; Johnson et al., 2015; Vonderheide et al., 2007). The remainder were produced in-house by standard hybridoma technology (Lob 7/8-m1, Lob 7/6-m1, Lob 8/2-m1, Lob 7/7-m1, and Lob 7/2-m1) or generated from patent sequences (24.2.1-m1; US patent 2009/0130715).

Figure 1A shows the dose-dependent proliferation of purified human CD40 transgenic (hCD40Tg) mouse B cells expressing (WT) or lacking (KO) mFcγRII in response to the three clinical mAbs. Lob 7/4-m1 and SGN40-m1 showed similar agonistic activity that was largely dependent upon crosslinking by the B cell FcγRII (Figure 1A). In contrast, CP870,893-m1 displayed much stronger agonistic activity, delivering two to three times more B cell proliferation with WT cells, and with much less dependence on crosslinking by mFcγRII.

Comparison of the remaining hCD40 mAbs in the B cell proliferation assay revealed a spectrum of activity (Figure 1B) with WT B cells (left bars) ranging from strong agonists (CP870,893-m1) to those with little or no activity (Lob 7/8-m1). When mFcγRII KO B cells were used (middle bars), only CP870,893-m1 continued to show strong activity (Figure 1B). However, importantly, when a feeder layer of CHO-K1 cells overexpressing hFcγRIIB (XL) was included in these cultures (White et al., 2011) all of the mAbs became highly agonistic, demonstrating their capacity to bind CD40 and provide potent agonism in the presence of non-physiological levels of mFcγRII-mediated crosslinking.

To compare *in-vivo* immunostimulatory activity, a vaccination model that measured SIINFEKL-specific CD8 (OT1) T cell responses against co-administered OVA in hCD40Tg mice was used (Figure 1C). The ability of the mAbs to expand OT1 cells correlated with their relative activity in the B cell proliferation assay, suggesting that similar rules dictate the agonistic activity of the mAbs on distinct cell types (B and T cells) and also *in vitro* and *in vivo*.

ChiLob 7/4, SGN40, and CP870,893 Engage Similar Epitopes in CRD1

The differences between mAbs in both the level of agonistic activity and mFcγRII dependency prompted us to examine more closely the role of the epitope in agonism. To precisely define the epitope bound by ChiLob 7/4 we determined the crystal

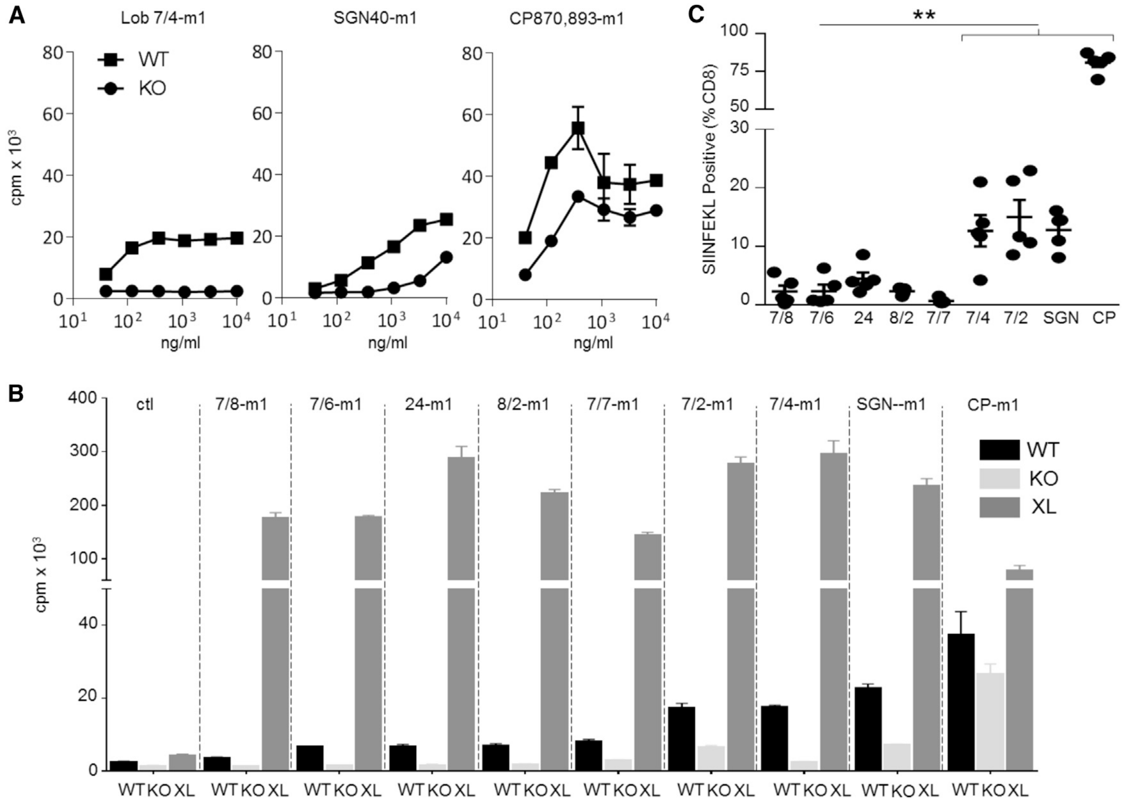

Figure 1. Mouse IgG1 Constant Regions Cannot Confer Agonistic Activity to all Anti-h CD40 mAbs

(A) Purified hCD40Tg (WT) or hCD40Tg/*Fcgr2b*$^{-/-}$ (KO) splenic mouse B cells were incubated with increasing concentrations of Lob 7/4-m1, SGN40-m1, or CP870,893-m1 for 4 days, and proliferation measured by [^{3}H]thymidine incorporation. Means ± SEM, n = 3, data representative of at least three experiments.

(B) Purified splenic WT or KO B cells in the absence or presence of irradiated hFcγRIIB-overexpressing CHO-K1 cells (XL) were treated as in (A) with 1 μg/mL each mAb (m1). Means ± SEM, n = 3, data representative of at least three experiments.

(C) 1 × 10^5 OTI cells were adoptively transferred to hCD40Tg mice 1 day before treatment with 30 μg of the indicated mAbs (m1). Phycoerythrin (PE)-labeled SIINFEKL+ cells, expressed as a percentage of total $CD8^+$ cells. Values for individual mice are shown; error bars represent means ± SEM.

Data representative of at least two experiments. Comparison of all CRD1-targeting mAbs individually with Lob 7/6-m1, **p < 0.01 using one-way ANOVA followed by Tukey's multiple comparisons test.

structure of its Fab' bound to a soluble CD40 extracellular domain (ECD) (Figure 2A; Table S1). Residues 1–211 of the ChiLob 7/4 light chain (LC) and 1–223 of the heavy chain (HC) were resolved (with the exception of residues 139–144 within CH1 of the HC). This leaves 3 terminal residues in the LC and an estimated 15 terminal residues in the HC unresolved in the electron density (see STAR Methods). CD40 was resolved in electron density for residues 21–120, corresponding to cysteine-rich domain 1 (CRD1), CRD2, and the majority of CRD3 (Figure 2A). The structure revealed that ChiLob 7/4 engaged an epitope at the N terminus of CD40 within CRD1 (Figure 2B). The complementarity-determining region (CDR) 3 of the ChiLob 7/4 HC formed interactions with CD40 giving the highest ΔG, as analyzed by PISA (Krissinel and Henrick, 2007), mostly within the A1 region of CRD1 (Figure 2B). The LC CDR1 and CDR3 contacted the B2 region of CRD1, while the CDR2 region contacted both the CRD1 A1 and B2 regions (Figure 2B).

Western blotting and competition experiments by surface plasmon resonance (SPR) and flow cytometry were used to map the binding epitopes of other hCD40 mAbs. ChiLob 7/4, SGN40, and CP870,893, all bound the full-length CD40 ECD, but not truncated forms where CRD1 was deleted (Figure 3A), and hence must recognize epitopes on CRD1 as reported previously for SGN40 and CP870,893 (Bjorck et al., 1994; Gladue et al., 2011). The mAb 24.2.1 bound to CD40 even when CRD1 was deleted, whereas Lob 7/6 bound when both CRD1 and CRD2 were deleted (Figure 3A). Flow cytometry data confirm domain-specific engagement as the CRD1-specific mAb Lob 7/4, SGN40, and CP870,893 all inhibited binding of Lob 7/4-fluorescein isothiocyanate (FITC) to hCD40Tg B cells, whereas Lob 7/6 and 24.2.1, which bind CRD domains closer to the membrane, did not (Figure 3B top panels). In contrast, Lob 7/6 and 24.2.1 blocked labeling of hCD40Tg B cells with Lob 7/6-FITC, whereas Lob 7/4, SGN40, and CP870,893 did not (Figure 3B, bottom panels). Furthermore, SPR showed that ChiLob 7/4, SGN40, and CP870,893 cross-competed with each other but not with Lob 7/6 for CD40 binding (Figure 3C). Thus, despite being associated with markedly different levels of agonistic activity (Figure 1), ChiLob 7/4, SGN40, and CP870,893 all bound to a similar region of CRD1 within CD40.

To differentiate the epitopes of ChiLob 7/4, SGN40, and CP870,893, we used a further deletion variant of CD40 in which

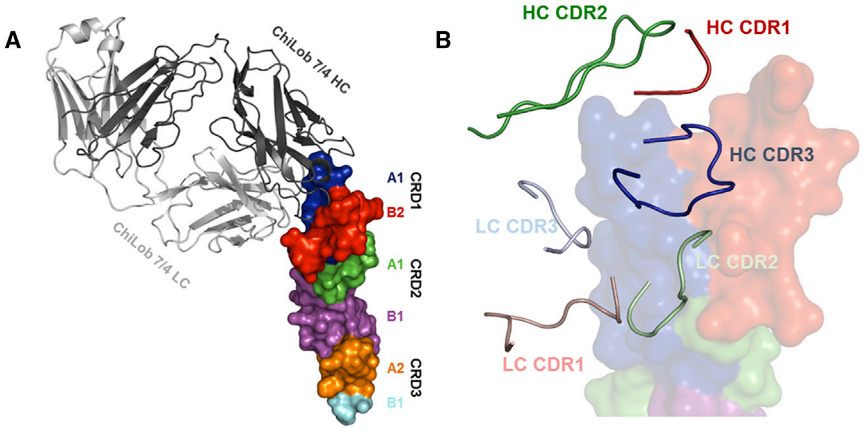

Figure 2. Crystal Structure of ChiLob 7/4 Fab' in Complex with CD40 Extracellular Domain

(A) The crystal structure of the CD40:ChiLob 7/4 Fab' complex shows the Fab' in gray (dark gray, heavy chain; light gray, light chain) and CD40 colored by CRD (blue, CRD1-A1; red, CRD1-B2; green, CRD2-A1; magenta, CRD2-B2; orange, CRD3-A1; cyan, CRD3-B2 [partial]).

(B) Interface, with structural elements removed for clarity. Shown are the CDR loops of ChiLob 7/4 in red for CDR1, green for CDR2, and blue for CDR3. Light color shades represent the light chain, dark shades the heavy chain. CD40 colored as in (A) (translucent). See also Table S1.

the A1 domain of CRD1 was removed (residues 23–37; Figure 2A). The binding of the mAbs to cells transfected with these variants was compared. ChiLob 7/4, SGN40, and CP870,893, which engage an epitope in CRD1, as well as 24.2.1 and Lob 7/6, which engage epitopes in CRD2-4, all bound approximately 80% of cells transfected with WT CD40 (Figure 3D). For the CD40-A1 deletion variant, 24.2.1 and Lob 7/6 still bound close to 80% of the cells, suggesting stable expression of this mutant. In contrast, binding of SGN40 was reduced to 58%, binding of ChiLob 7/4 was reduced to 9%, and binding of CP870,893 was completely lost (Figure 3D). These data reflect an epitope variation for these mAbs. In a simple modeling exercise based on the determined crystallographic structure of ChiLob 7/4, we demonstrate how these differences can be rationalized (Figure S1A). The modeling suggests that an interaction with CRD1 is lost while another interaction with CRD2 is gained, placing the binding epitope for SGN40 further down the CD40 scaffold compared with the one seen in the structure of ChiLob 7/4:CD40.

Western blotting and flow cytometry were used to produce epitope maps for a further six anti-hCD40 mAbs (Lob 7/2, Lob 7/4, Lob 7/6-Lob 7/7, Lob 7/8, Lob 8/2, and 24.2.1; Figures S1B and S1C). The proposed distribution of these epitopes in relation to the CD40 domain structure is presented in Figure 3E.

Surface alanine scanning mutagenesis was used to confirm and refine these observations (Figure 4). CHO-K1 cells stably transfected with CD40 proteins in which consecutive pairs of surface amino acids were mutated to alanine were used to define residues critical for mAb binding. Consistent with the data in Figure 3, residues R27 and E28 close to the N terminus of CD40 were found to be essential for CP870,893 binding, whereas ChiLob 7/4 was dependent upon residues F54 and T55 within the B2 domain of CRD1 (Figure 4). SGN40 and Lob 7/2 bound epitopes related to that of ChiLob 7/4, being additionally dependent upon residues E56 and T57 (Figure 4). This technique also identified residues in CRD2 important for the binding of Lob 8/2 (G63 × E64 K94 G95) and Lob 7/7 (D84 P85) (Figure 4). Overall these data gave a detailed positional map of the binding sites on extracellular CD40 for each of the antibodies.

When the defined epitope locations are correlated with agonistic activity (Figure 1), it is clear that, without extensive crosslinking, only those mAbs that bind to CRD1 are strong agonists. The inability of mAbs that bind epitopes in CRD2-4 to agonize CD40 may reflect reduced availability of their Fc regions to engage FcγR expressed at physiological levels. This notion was supported by experiments that demonstrated CRD2-4 binding mAbs were also less able to direct macrophage-mediated phagocytosis of opsonized B cells compared with CRD1 binders, a process that also requires FcγR interaction (Figure S2), as well as the strong agonism displayed by all antibodies when crosslinking was forced by super-physiological levels of mFcγRII (Figure 1B).

The Influence of Human Isotype on Epitope-Dependent Agonism

Having established the role of epitope in the context of the m1 isotype, we next explored the influence of epitope specificity in the context of human IgG isotypes, h1 and h2. As demonstrated previously (White et al., 2015), ChiLob 7/4-h2 and SGN40-h2 (mAbs expressed with h2 constant domains) caused proliferation of isolated hCD40Tg mouse B cells, whereas their h1 counterparts were largely inactive (Figure 5A).

The activity of CP870,893 was largely independent of isotype, with both h1 and h2 formats driving robust B cell expansion (Figure 5A). Moreover, the overall level of agonistic activity observed with CP870,893 was higher than either ChiLob 7/4-h2 or SGN40-h2, with >4- to 30-fold greater B cell proliferation at 1 μg/mL depending upon the isotype and consistent with clinical studies demonstrating an approximately 10-fold lower maximum tolerated dose for CP870,893 versus SGN40 or ChiLob 7/4 (Hussein et al., 2010; Johnson et al., 2015; Vonderheide et al., 2007). The increased agonistic activity of CP870,893 was not due to enhanced CD40 binding as similar amounts of each mAb bound to B cells over the concentration range analyzed (data not shown). This is further indication of the different mode of action of CP870,893.

Similar differences were observed in the ability of the different mAbs to activate isolated human B cells, as assessed by CD23 upregulation (Figure 5B). In addition, all h1 and h2 mAbs activated human B cells equivalently when cells expressing super-physiological levels of hFcγRIIB were added (Figure 5B), suggesting similar mechanisms of CD40 activation in mouse and human B cells.

We next extended the panel of h1 and h2 hCD40 mAbs to investigate how epitope specificity influenced agonism

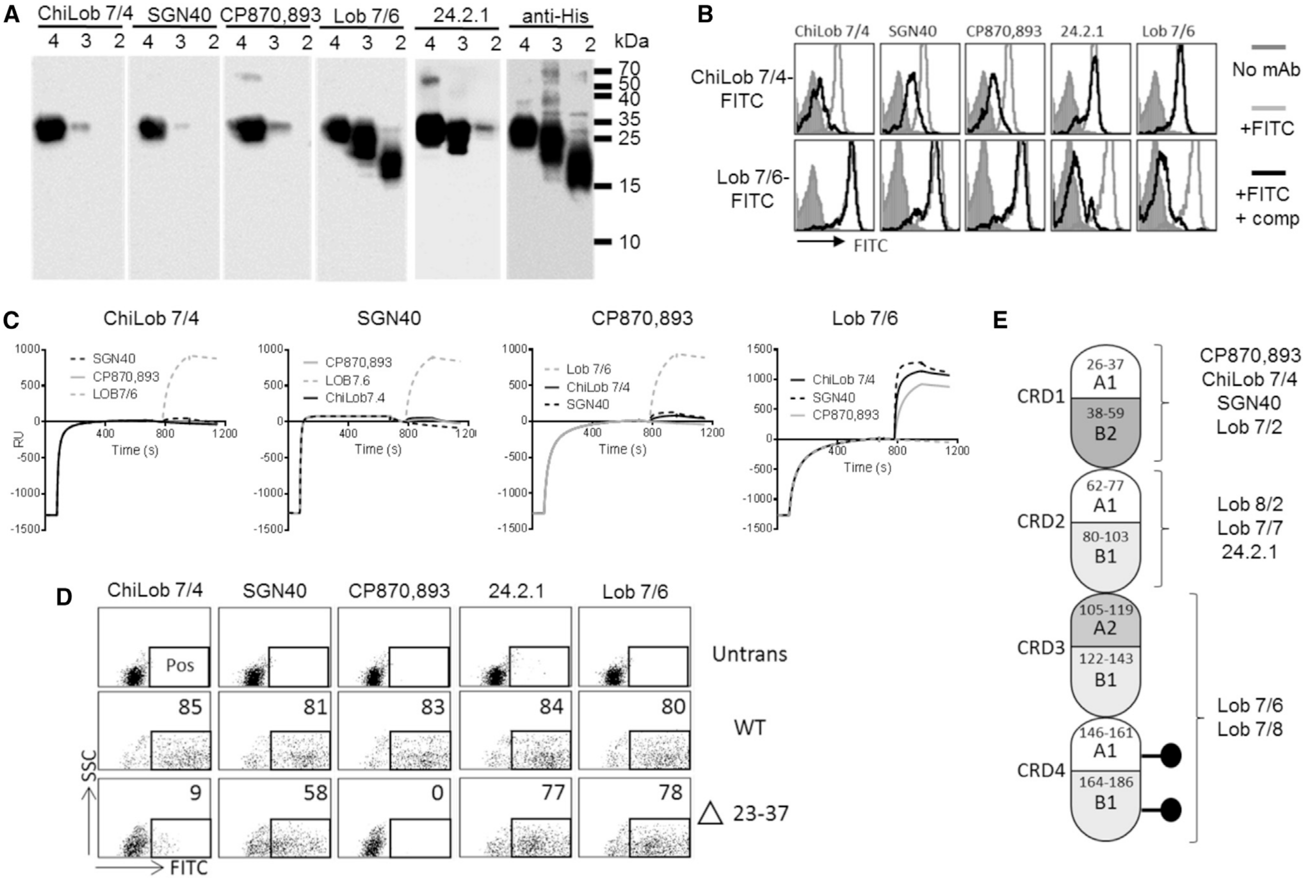

Figure 3. Epitope Mapping of Anti-hCD40 mAbs

(A) C-terminally His-tagged CD40 proteins consisting of 4, 3, or 2 CRD domains (4 = full-length; 3 = CRD1 deleted; 2 = CRD1 and CRD2 deleted) were analyzed by western blotting with the mAbs indicated above each panel used for detection. A composite image from multiple blots with different antibodies is shown.

(B) hCD0Tg mouse B cells were incubated with FITC-labeled Lob 7/4 or Lob 7/6, as indicated, in the absence or presence of a 10-fold excess of competitor (comp) mAbs indicated above each plot. Cell labeling was assessed by flow cytometry; filled histogram, unlabeled cells; light gray, FITC-labeled mAbs alone; black line, FITC-labeled mAbs + competitor.

(C) Surface plasmon resonance analysis of mAb competition for CD40 binding. The mAbs indicated above each plot were flowed over immobilized hCD40 ECD for 800 s to allow saturation of hCD40 binding sites, followed by the addition of individual competitor mAb for 350 s. Non-competitive mAbs (i.e., mAbs without overlapping epitope) give a response greater than 500 response units (RU).

(D) Untransfected 293 cells (none), or cells transfected with full-length (WT) human CD40 or CD40 in which N-terminal amino acids 23–37 had been deleted, were incubated with the indicated anti-hCD40 mAbs (h2 isotype). Bound mAbs were detected with anti-human Fc-FITC. The percentage of cells in the boxed region, denoting positive staining, is shown for each plot. Results from one of two experiments shown.

(E). Schematic of the approximate locations of epitopes for nine human CD40 mAbs analyzed. See also Figure S1.

(Figure 5C). As with m1, we found that B cell agonistic activity was largely confined to those mAbs binding to the CRD1 membrane distal domain. Lob 8/2 was the exception in that it bound to CRD2 but was agonistic, at least as h2. For all agonists, h2 was the most active of the human isotypes, particularly in the case of Lob 7/2, SGN40, and Lob 7/4, where h2 and m1 showed very similar agonism with h1 less active. However, the most striking difference between isotypes was seen when using mFcγRII KO B cells where, in almost all cases, h1 and m1 completely lost activity, while h2 retained agonistic function. An exception was the low level of agonism observed with Lob 7/2 h1, which was retained in the absence of mFcγRII. This is consistent with previous findings that h2 agonistic activity is independent of FcγR interaction (White et al., 2015). Finally, CP870,893 was again shown to be unique in evoking very high agonistic activity irrespective of isotype, and in retaining appreciable activity in the absence of mFcγRII. This distinct isotype insensitivity, compared with other mAbs such as ChiLob 7/4, was recapitulated in nuclear factor κB activation assays (Figure S3).

To extend these studies *in vivo* we performed adoptive transfer of OT1 cells into hCD40Tg mice (Figure 5D). As with the m1 of these reagents (Figure 1), the *in vitro* and *in vivo* data corresponded closely. ChiLob 7/4-h2 and SGN40-h2 stimulated strong expansion of OT1 T cells, whereas h1 was significantly less active (Figure 5D). This was particularly striking for SGN40 where, compared with h2, h1 showed very little activity. Importantly, and unlike m1 (Figure 1C), the activity of ChiLob 7/4-h2 and SGN40-h2 was independent of mFcγRII, showing comparable activity in mFcγRII KO mice, and confirming previous observations where F(ab')$_2$ fragments of ChiLob 7/4 h2 demonstrated

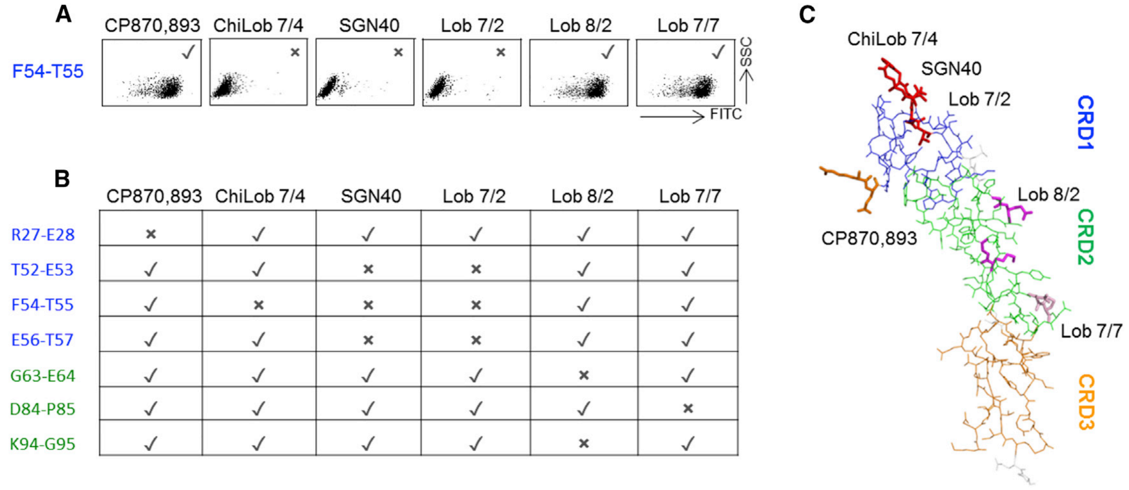

Figure 4. Alanine Scanning Epitope Mapping of Anti-hCD40 mAbs

CHO-k1 cells stably expressing mutant hCD40 proteins were incubated with the indicated m1 mAb and binding detected by flow cytometry with anti-mouse Fc-FITC.

(A) Example plots for the F54-T55 mutant to illustrate positive (tick) and negative (cross) binding results.

(B) Summary of data for additional hCD40 mutants (at least two experiments per combination).

(C) Deduced mAb epitopes are illustrated on the hCD40 crystal structure resolved in Figure 2, and are color coded with those for ChiLob 7/4, SGN40, and Lob 7/2 overlapping. See also Figure S2.

wholly FcγR-independent activity *in vivo* (White et al., 2015). In comparison, CP870,893 stimulated greater expansion of OT1 T cells as h1 or h2 compared with SGN40 and ChiLob 7/4 in the presence or absence of FcγRII. Thus, in contrast to ChiLob 7/4 and SGN40, CP870,893 displayed much greater agonistic activity both *in vitro* and *in vivo*, which was largely independent of isotype and mFcγRII engagement. Overall these data support the notion that mAbs binding to epitopes closer to the cell membrane make weaker agonists, irrespective of how agonism is achieved, either through the h2 isotype or FcγRII engagement.

The unusual nature of CP870,893 was underlined when we examined the effect of different h2 isoforms on its agonistic activity. Previously we showed that the FcγR-independent agonistic activity of ChiLob 7/4-h2 was dependent on the h2B isoform, which exists in unmodified h2 preparations when hinge region disulfide bonds are "shuffled" (White et al., 2015). The h2A and h2B forms can be enriched by chemical skewing in different redox buffers or by introducing cysteine to serine mutations, which "lock" the molecule in different conformations. Figure 6 shows that, similar to Lob 7/4 (White et al., 2015), the agonistic activity of the SGN40 was stronger both *in vitro* and *in vivo* when enriched as h2B versus h2A (Figures 6A–6C). In contrast, CP870,893 was equally active as h2A or h2B produced through either skewing or mutagenesis (Figure 6A), both *in vitro* (Figure 6B) and *in vivo* (Figure 6C). These results underline the unusual ability of CP870,893 to retain agonistic activity in all isotypes and isoforms and without FcγR crosslinking.

mAbs that Engage CRD2-4 Block CD40L and Are Antagonists

Having established the factors regulating potent agonism, we next examined the requirements for antagonism in our panel of mAbs by assessing their ability to inhibit CD40L-mediated B cell activation. Flow cytometry demonstrated that none of the mAbs that engage CRD1 (ChiLob 7/4, SGN40, CP870,893, or Lob 7/2) prevented binding of CD40L to hCD40Tg mouse B cells (Figure 7A), while mAbs that bound epitopes in CRD2-4 either partially (Lob 7/7) or completely (Lob 7/7, Lob 7/8, 24.2.1, and Lob 8/2) blocked CD40L binding. The structural superposition of our ChiLob 7/4:CD40 complex with the CD40:CD40L complex (PDB: 3QD6) demonstrates that ChiLob 7/4 and the CD40L bind to different CRDs on opposite faces of CD40 (Figure 7B). Since ChiLob 7/4, SGN40, and CP870,893 compete for related epitopes in CRD1 (Figures 3 and 4), none of these mAbs would be predicted to block the CD40-CD40L interaction. mAbs that engage epitopes in CRD2-4 may, however, interfere with CD40L binding.

The ability to block CD40-CD40L interaction correlated with differences in antagonistic function. When added to hCD40Tg B cells, hexameric mCD40L, a form with potent agonistic properties (Haswell et al., 2001), activated the cells as measured by homotypic adhesion (Figure 7C, top panels), upregulation of CD23, CD86, and MHCII (Figure 7C, lower three panels), and enhanced B cell proliferation (Figure 7D). When m1 mAbs binding CRD1 (ChiLob 7/4, SGN40, CP870,893, and Lob 7/2) were added together with CD40L, B cell activation (Figure 7C) and proliferation (Figure 7D) were increased compared with CD40L alone. In contrast, Lob 7/7, which partially blocks CD40L binding, had no significant additive effect on CD40L-mediated B cell activation or proliferation (Figures 7C and 7D), whereas Lob 8/2, Lob 7/8, and 24.2.1, which all prevent CD40-CD40L interaction inhibited B cell activation and division compared with CD40L alone (Figures 7C and 7D).

Similar results were observed when h2 mAbs were used (Figure 7E). Lob 7/4 and Lob 7/2 (CRD1 binders) were both agonistic in the absence of exogenous ligand and maintained robust B cell

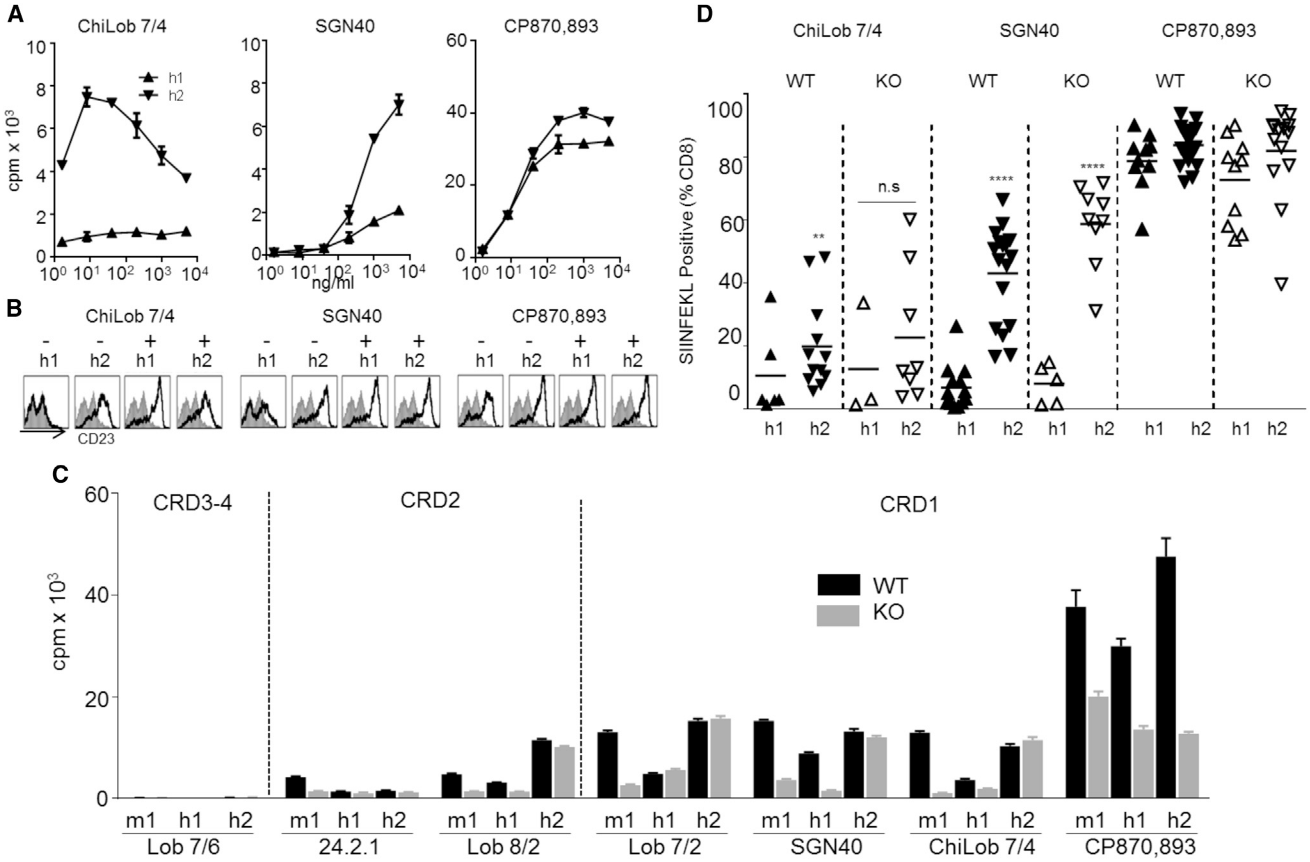

Figure 5. Influence of Human IgG Isotype on Epitope-Dependent Agonism

(A) Purified hCD40Tg mouse splenic B cells were incubated with the indicated mAb isotypes at various concentrations for 4 days, and proliferation was measured as in Figure 1A. Means ± SEM, n = 3, data representative of at least three experiments.

(B) Purified human B cells were incubated with the indicated mAbs at 1 μg/mL in the absence (−) or presence (+) of crosslinking cells overexpressing hFcγRIIB, as in Figure 1B. Histograms show expression of CD23 (black lines) compared with untreated cells (gray histograms) as determined by flow cytometry.

(C) Purified hCD40Tg WT or KO mouse splenic B cells were treated as in (A) with 1 μg/mL of each mAb of m1, h1, or h2 isotype, and proliferation determined by [^{3}H]thymidine incorporation. Means ± SEM, n = 5, data representative of at least three experiments.

(D) 1×10^5 OT1 cells were adoptively transferred to hCD40Tg mice 1 day before treatment with 100 μg h1 or h2 of the indicated mAbs and 100 μg OVA. OT1 cells in peripheral blood were quantified on day 5 using PE-labeled SIINFEKL tetramer, expressed as percentage of total CD8$^+$ cells. Data points represent individual animals from at least two independent experiments per mAb. Horizontal bars indicate mean values.

**$p < 0.001$, ****$p < 0.0001$, h1 versus h2 of same mAbs using the Student's unpaired t test. See also Figure S3.

proliferation in its presence (Figure 7E). In contrast, 24.2.1 and Lob 7/6, which engage epitopes in CRD2-4 and compete for CD40L binding, were not agonistic alone and prevented activation by CD40L (Figure 7E). An exception, as noted for Figure 5C, was Lob 8/2, which, despite blocking CD40L interaction, enhanced B cell proliferation in both the presence and absence of CD40L when used as h2.

Collectively, our data suggest that agonistic hCD40 mAbs do not compete with CD40L binding, and retain agonistic activity in its presence. In contrast, antagonistic anti-hCD40 mAbs bind to CRD2-4 that are closer to the cell membrane and tend to block CD40L binding, and thus antagonize CD40L activity.

DISCUSSION

Here, we performed a comprehensive analysis of the roles that epitope specificity and isotype play in mediating hCD40 mAb function. Using a panel of nine hCD40 mAbs, including three in clinical development, engaging epitopes throughout the length of CD40 and with a number of different IgG isotype frameworks, we reveal a complex interplay between epitope location and isotype that produces a range of activities from antagonism to strong agonism. These insights provide the opportunity to fine-tune hCD40 mAb function for defined levels of activity, as may be required for specific tissue locations or therapeutic circumstances.

Both epitope specificity (Barr and Heath, 2001; Bjorck et al., 1994; Ellmark et al., 2002; Malmborg Hager et al., 2003; Yamniuk et al., 2016) and isotype (Dahan et al., 2016; Li and Ravetch, 2011; White et al., 2011, 2013, 2014, 2015) have separately been shown to regulate hCD40 mAb function. However, how these factors combine to mediate the wide-ranging levels of activity had not been systematically addressed. Mouse IgG1 constant regions can promote CD40 mAb agonism through

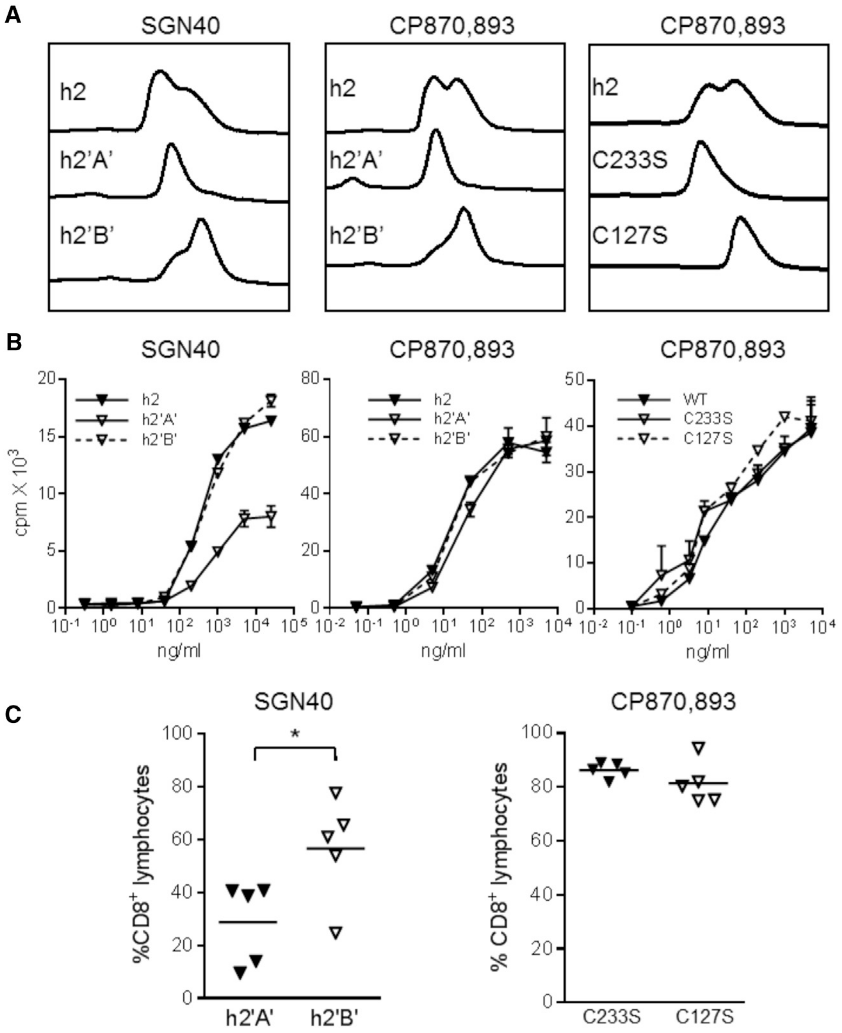

Figure 6. Influence of Hinge Conformation on SGN40 and CP870,893 Agonistic Activity

(A) CE-SDS profiles of the mAbs chemically skewed toward their h2A and h2B conformations (h2′A′ and h2′B′, respectively) or "locked" in h2A (through point mutation C233S) and h2B (through point mutation C127S).

(B) Ability of the different h2 mAbs to stimulate proliferation of hCD40Tg mouse B cells was analyzed as described in Figure 1A. Data are presented as means ± range of duplicate samples.

(C) The ability of the mAb to stimulate OT1 cell expansion *in vivo* was analyzed as described for Figure 1C. Results for individual animals are plotted. Horizontal bars indicate mean values.

*$p < 0.05$ for h2′A′ versus h2′B′ using the Student's unpaired t test.

interaction with FcγRIIB mediating mAb crosslinking *in vitro* and *in vivo* (White et al., 2011). However, when our nine hCD40 mAbs were expressed with the same m1 backbone, a range of activities was observed, from non-agonistic to strongly agonistic, clearly demonstrating that m1 constant regions cannot confer stimulatory activity to anti-hCD40 mAbs per se. Indeed, only those mAbs engaging epitopes in CRD1 (ChiLob 7/4, SGN40, CP870,893, and Lob 7/2) were agonistic as m1, whereas those that bound CRD2-4 were not, demonstrating an essential role for epitope location.

Protein crystallography elucidated the precise epitope for ChiLob 7/4; close to the N terminus of CD40, most distal from the cell membrane and on the opposite face of CD40 to the CD40L binding site. We propose that the dependence on membrane-distal CRD1 binding epitopes for m1-mediated agonistic activity reflects the requirement for the mAb Fc to engage FcγRII on adjacent cells (White et al., 2013). Whereas the Fc domains of mAbs that bind CRD1 may be accessible to FcγRII, steric constraints may prevent optimal FcγR engagement for mAbs that bind epitopes closer to the membrane. This hypothesis is supported by the similar dependence on hCD40 mAb epitope location for macrophage phagocytosis of B cells that is dependent upon activatory FcγR engagement. Dahan et al. (2016) recently reported that the hCD40 mAb clone 40.1, which engages an epitope toward the N terminus of CD40, induced greater T cell responses in hCD40Tg mice than clone 40.2, which binds further down the molecule and competes with CD40L for CD40 association. Introduction of Fc mutations to selectively enhance hFcγRIIB affinity increased the stimulatory activity of both clones; however, the activity of 40.1 remained substantially greater even in this context (Dahan et al., 2016). In the current study, all m1 mAbs, regardless of epitope location, became highly agonistic *in vitro* when crosslinking was forced through introduction of FcγRIIB hyper-expressing accessory cells, reminiscent of earlier studies by Pound et al. (1999) and, more recently, Yamniuk et al. (2016). These data suggest that, for mAbs whose agonism is dependent upon FcγRIIB binding, activity level is governed by a combination of three factors: (1) accessibility of the mAb Fc (determined by epitope location); (2) affinity of the mAb Fc for FcγRIIB; and (3) level of FcγRIIB expression in the local microenvironment.

A similar relationship with epitope location was observed when agonistic activity was examined in the context of h2 constant regions that can confer FcγR-independent agonistic activity due to a unique arrangement of hinge disulfides (White et al., 2015). Similar to results with m1, h2 mAbs that engaged epitopes in CRD1 were agonistic, whereas two of three h2 mAbs that engaged epitopes in CRD2-4 did not display agonistic function. The exception was Lob 8/2, which bound an epitope in CRD2 and agonized CD40 when expressed as h2 but not when expressed as m1. These results suggest that the relationships between epitope and agonistic activity are distinct for m1 and h2. Exactly how h2 anti-hCD40 mAbs mediate CD40 activation in the absence of FcγR binding is not known, and the subject of our ongoing research. Under physiological conditions, CD40 is activated through engagement by trimeric CD40L. Although trimerization is believed to be required for activation, CD40 is

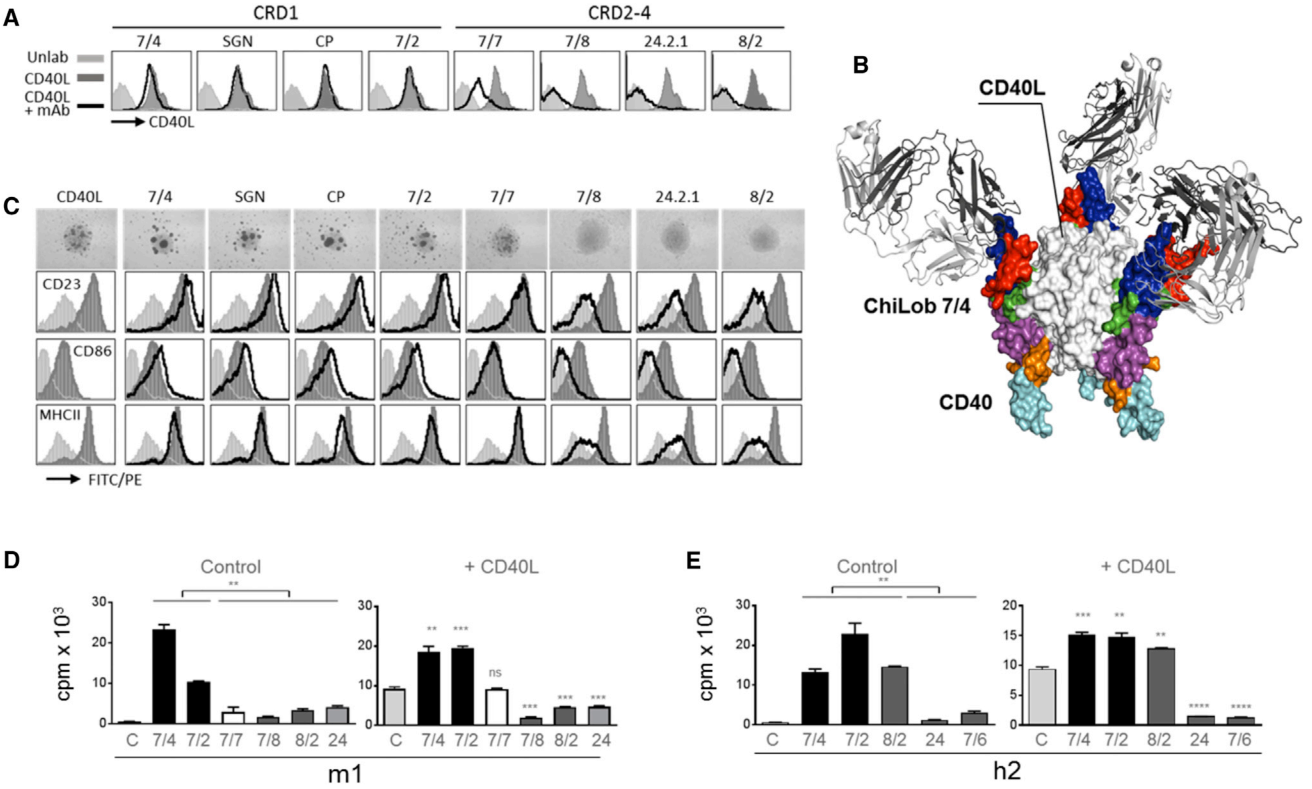

Figure 7. Antagonist Anti-hCD40 mAbs Engage CRD2-4 Domains

(A) Purified hCD40Tg mouse splenic B cells were incubated with or without the indicated mAb, all of m1 isotype, followed by hexameric mCD40L-h1Fc. Bound CD40L was then detected with anti-human-FITC and flow cytometry.

(B) Structure superposition of CD40:Lob 7/4 Fab' complex (colored as in Figure 2A) with the CD40:CD40L complex (PDB: 3QD6). CD40L is shown as a trimer binding to the opposite side of CD40 than Lob 7/4 and to a different CRD (CD40L shown as white surface representation).

(C) Purified hCD40Tg mouse splenic B cells were incubated with CD40L alone (10 μg/mL) or together with the indicated mAbs (m1 at 1 μg/mL) for 20 hr. Activation was assessed by homotypic adhesion (top) or flow cytometry to assess upregulation of activation markers. Results from one of at least three experiments for each mAb are shown.

(D) Purified hCD40Tg mouse splenic B cells were incubated with the indicated mAbs (m1 at 1 μg/mL) under control conditions (left panel) or in the presence of CD40L (10 μg/mL, right panel). Proliferation was assessed by [^{3}H]thymidine incorporation as in Figure 1A and plotted as means ± SEM.

(E) As in (D), except that h2 mAbs were used. Means ± SEM of triplicate samples representing results from two or three experiments per mAb.

**p < 0.01, ***p < 0.001, ****p < 0.0001 using the unpaired Student's t test, left hand plots for indicated groups, right hand plots versus CD40L alone.

thought to exist as pre-formed dimers or trimers in membranes of unstimulated cells (Chan et al., 2000; Smulski et al., 2013). Elegant studies using recombinant CD40L molecules of different stoichiometries have also demonstrated that higher-order clustering of CD40 can enhance signal intensity to levels above those observed with the trimeric ligand (Graves et al., 2014; Haswell et al., 2001). We hypothesize that h2B hCD40 mAbs are compact, and when they engage epitopes in CRD1 they are able to cluster pre-formed CD40 dimers or trimers in the membrane to promote signaling in the absence of additional cross-linking. This is similar to a model proposed for the agonistic death receptor 5 mAb AMG 655 where co-crystallization of receptor-ligand-AMG 655 complexes suggest the formation of higher-order signaling complexes in the cell membrane (Graves et al., 2014). It is possible that mAbs engaging epitopes in CRD2-4 disrupt pre-formed complexes and prevent this mechanism of activation. Consistent with this hypothesis, we found that CRD1 binding mAbs retained B cell activation in response to CD40L, whereas those that engage CRDs 2–4 inhibit CD40L-mediated activation, as has been reported previously (Challa et al., 1999; Pound et al., 1999). Further studies will be required to delineate the precise mechanism of CD40 activation by h2 mAbs. Importantly, however, and similar to m1, the presence of mFcγRII-overexpressing cells rendered all h2 anti-hCD40 mAbs highly agonistic, regardless of epitope, consistent with the ability of hyper-crosslinking to force activation through non-physiological receptor clustering.

Dahan et al. (2016) recently demonstrated that the relative agonistic activity of h1 and h2 hCD40 mAbs in hCD40Tg mice is influenced by the repertoire of FcγR available for Fc crosslinking; in mice expressing all human FcγR, h1 versions of CP870,893 and Lob 7/4 became more active than their h2 counterparts due to the superior ability of h1 to engage human FcγRIIB. Previous studies have similarly demonstrated that the isotype dependence of hCD40 mAbs is influenced by the local FcγR availability (White et al., 2011; Wilson et al., 2011). However, Dahan et al. also proposed that FcγR crosslinking was a requirement for the agonistic activity of h2 anti-hCD40 mAbs,

because deglycosylated CP870,893, with its reduced ability to engage FcγR (Tao and Morrison, 1989), lost activity *in vivo* (Dahan et al., 2016). This contrasts with our own work (White et al., 2015) as well as that of Richman and Vonderheide (2014). Those authors demonstrated that $F(ab')_2$ fragments of CP870,893, which *de facto* cannot engage FcγR, could activate human B cells to a similar extent to the parental h2 IgG *in vitro*, similar to our own studies with ChiLob 7/4 that demonstrated strong *in vivo* agonistic activity of h2 (but not h1) $F(ab')_2$ fragments in stimulating T cell responses against co-administered OVA (White et al., 2015). The present study clearly shows FcγR-independent agonistic activity of multiple h2 anti-hCD40 mAbs (SGN40, CP870,893, Lob 7/2, and Lob 8/2) *in vitro* using FcγR-deficient B cells, as well as FcgRIIB-independent *in vivo* activity.

While some general rules regarding the roles of epitope and isotype in anti-hCD40 mAb function can be imputed from this study and used to aid design of future therapeutic mAbs with defined characteristics, it is clear that this relationship is not simple and that other factors can influence activity. Multiple hCD40 mAbs are in early clinical development and it will be important to elucidate the characteristics of each of these agents to optimize activity. The different agonistic mechanisms exhibited (m1 versus h2 versus isotype-independent activity of CP870,893) will also need to be investigated for differences in therapeutic efficacy and toxicity. CD40 is just one member of the TNFR superfamily, many others of which are also targets for therapeutic mAb development. Whether the rules revealed for CD40 will apply to these other targets that exhibit similarities in their mechanisms of activation, also remains to be seen. Other studies have shown that epitope location plays a crucial role in the activity of mAbs against the Ig family member CD28, with epitopes in the lateral C''D loop imparting "super-agonistic" properties enabling T cell activation in the absence of TCR stimulation (Luhder et al., 2003). The mechanisms responsible are distinct from those involved in CD40 activation, but further highlight the complexities involved in developing agonistic mAb therapeutics compared with those designed to block receptor-ligand interactions.

As well as helping to design effective immunotherapeutics, the different properties exhibited by the range of hCD40 mAbs in this study also provides a valuable toolbox with which to probe the biology of TNFR activation.

STAR★METHODS

Detailed methods are provided in the online version of this paper and include the following:

- KEY RESOURCES TABLE
- CONTACT FOR REAGENT AND RESOURCE SHARING
- EXPERIMENTAL MODEL AND SUBJECT DETAILS
  - Mice
  - Human Samples
- METHOD DETAILS
  - Antibodies and Reagents
  - B Cell Activation and Proliferation
  - Immunisation and Assessment of Immune Responses
  - Generation and Expression of CD40 Constructs
  - Protein Crystallography
  - NF-κB Assay
  - Surface Plasmon Resonance
  - Phagocytosis Assay
- QUANTIFICATION AND STATISTICAL ANALYSIS
- DATA AND SOFTWARE AVAILABILITY

SUPPLEMENTAL INFORMATION

Supplemental Information includes three figures and one table and can be found with this article online at https://doi.org/10.1016/j.ccell.2018.02.009.

ACKNOWLEDGMENTS

We would like to thank colleagues from the Cancer Sciences Unit who provided advice and technical support, the Biomedical Research Facility for animal husbandry, and Professor Rienk Offringa for technical discussion. We thank Chris Holes at the Macromolecular Crystallisation Facility, Biological Sciences, and staff at the European Synchrotron Radiation Facility for access and excellent user support, and acknowledge support through MX1732 and MX1848. Funding was provided by CRUK grants C1477/A10834, C1477/A20537, C34999/A18087, and C328/A25139 as well as EU FP7 grant 602262-2.

AUTHOR CONTRIBUTIONS

X.Y. designed and performed the experiments, analyzed and interpreted data, and helped write the manuscript. H.T.C., C.O., I.T., O.D., S.B., L.N.D., P.D., L.R.D., C.P., L.O.B., C.I.M., and R.R.F. generated or provided key reagents or performed and analyzed the research. A.R.P. supervised data collection, analyzed data, and edited the manuscript. I.T. performed crystallographic structure analysis, discussed and interpreted data, and wrote the manuscript. M.S.C. supervised data collection, discussed and interpreted data, and edited the manuscript. M.J.G. and A.W. designed the study, analyzed and interpreted data, and wrote the manuscript.

DECLARATION OF INTERESTS

Mark S. Cragg acts as a consultant for BioInvent and has received research funding from BioInvent, GSK, iTeos, and Roche. Martin J. Glennie acts as a consultant to a number of biotech companies and receives institutional payments and royalties from antibody patents and licenses. This work is related to patent Family WO 2015/145360 protecting antibodies containing modified IgG2 domains which elicit agonist or antagonistic properties.

Received: October 24, 2017
Revised: December 20, 2017
Accepted: February 15, 2018
Published: March 22, 2018

REFERENCES

Ahonen, C., Manning, E., Erickson, L.D., O'Connor, B., Lind, E.F., Pullen, S.S., Kehry, M.R., and Noelle, R.J. (2002). The CD40-TRAF6 axis controls affinity maturation and the generation of long-lived plasma cells. Nat. Immunol. *3*, 451–456.

Allen, M.J., Guo, A., Martinez, T., Han, M., Flynn, G.C., Wypych, J., Liu, Y.D., Shen, W.D., Dillon, T.M., Vezina, C., and Balland, A. (2009). Interchain disulfide bonding in human IgG2 antibodies probed by site-directed mutagenesis. Biochemistry *48*, 3755–3766.

Barr, T.A., and Heath, A.W. (2001). Functional activity of CD40 antibodies correlates to the position of binding relative to CD154. Immunology *102*, 39–43.

Beatty, G.L., Chiorean, E.G., Fishman, M.P., Saboury, B., Teitelbaum, U.R., Sun, W., Huhn, R.D., Song, W., Li, D., Sharp, L.L., et al. (2011). CD40 agonists alter tumor stroma and show efficacy against pancreatic carcinoma in mice and humans. Science *331*, 1612–1616.

Beers, S.A., Glennie, M.J., and White, A.L. (2016). Influence of immunoglobulin isotype on therapeutic antibody function. Blood *127*, 1097–1101.

CellPress

Bjorck, P., Braesch-Andersen, S., and Paulie, S. (1994). Antibodies to distinct epitopes on the CD40 molecule co-operate in stimulation and can be used for the detection of soluble CD40. Immunology *83*, 430–437.

Boross, P., Arandhara, V.L., Martin-Ramirez, J., Santiago-Raber, M.L., Carlucci, F., Flierman, R., van der Kaa, J., Breukel, C., Claassens, J.W., Camps, M., et al. (2011). The inhibiting Fc receptor for IgG, FcgammaRIIB, is a modifier of autoimmune susceptibility. J. Immunol. *187*, 1304–1313.

Bulek, A.M., Madura, F., Fuller, A., Holland, C.J., Schauenburg, A.J., Sewell, A.K., Rizkallah, P.J., and Cole, D.K. (2012). TCR/pMHC optimized protein crystallization screen. J. Immunol. Methods *382*, 203–210.

Challa, A., Pound, J.D., Armitage, R.J., and Gordon, J. (1999). Epitope-dependent synergism and antagonism between CD40 antibodies and soluble CD40 ligand for the regulation of CD23 expression and IgE synthesis in human B cells. Allergy *54*, 576–583.

Chan, A.C., and Carter, P.J. (2010). Therapeutic antibodies for autoimmunity and inflammation. Nat. Rev. Immunol. *10*, 301–316.

Chan, F.K., Chun, H.J., Zheng, L., Siegel, R.M., Bui, K.L., and Lenardo, M.J. (2000). A domain in TNF receptors that mediates ligand-independent receptor assembly and signaling. Science *288*, 2351–2354.

Croft, M., Benedict, C.A., and Ware, C.F. (2013). Clinical targeting of the TNF and TNFR superfamilies. Nat. Rev. Drug Discov. *12*, 147–168.

Dahan, R., Barnhart, B.C., Li, F., Yamniuk, A.P., Korman, A.J., and Ravetch, J.V. (2016). Therapeutic activity of agonistic, human anti-CD40 monoclonal antibodies requires selective fcgammar engagement. Cancer Cell *29*, 820–831.

Dillon, T.M., Ricci, M.S., Vezina, C., Flynn, G.C., Liu, Y.D., Rehder, D.S., Plant, M., Henkle, B., Li, Y., Deechongkit, S., et al. (2008). Structural and functional characterization of disulfide isoforms of the human IgG2 subclass. J. Biol. Chem. *283*, 16206–16215.

Ellmark, P., Ottosson, C., Borrebaeck, C.A., Malmborg Hager, A.C., and Furebring, C. (2002). Modulation of the CD40-CD40 ligand interaction using human anti-CD40 single-chain antibody fragments obtained from the n-CoDeR phage display library. Immunology *106*, 456–463.

Emsley, P., Lohkamp, B., Scott, W.G., and Cowtan, K. (2010). Features and development of Coot. Acta Crystallogr. D Biol. Crystallogr. *66*, 486–501.

Evans, P.R., and Murshudov, G.N. (2013). How good are my data and what is the resolution? Acta Crystallogr. D Biol. Crystallogr. *69*, 1204–1214.

Gladue, R.P., Paradis, T., Cole, S.H., Donovan, C., Nelson, R., Alpert, R., Gardner, J., Natoli, E., Elliott, E., Shepard, R., and Bedian, V. (2011). The CD40 agonist antibody CP-870,893 enhances dendritic cell and B-cell activity and promotes anti-tumor efficacy in SCID-hu mice. Cancer Immunol. Immunother. *60*, 1009–1017.

Graves, J.D., Kordich, J.J., Huang, T.H., Piasecki, J., Bush, T.L., Sullivan, T., Foltz, I.N., Chang, W., Douangpanya, H., Dang, T., et al. (2014). Apo2L/TRAIL and the death receptor 5 agonist antibody AMG 655 cooperate to promote receptor clustering and antitumor activity. Cancer Cell *26*, 177–189.

Grewal, I.S., and Flavell, R.A. (1998). CD40 and CD154 in cell-mediated immunity. Annu. Rev. Immunol. *16*, 111–135.

Malmborg Hager, A.C., Ellmark, P., Borrebaeck, C.A., and Furebring, C. (2003). Affinity and epitope profiling of mouse anti-CD40 monoclonal antibodies. Scand. J. Immunol. *57*, 517–524.

Haswell, L.E., Glennie, M.J., and Al-Shamkhani, A. (2001). Analysis of the oligomeric requirement for signaling by CD40 using soluble multimeric forms of its ligand, CD154. Eur. J. Immunol. *31*, 3094–3100.

Hussein, M., Berenson, J.R., Niesvizky, R., Munshi, N., Matous, J., Sobecks, R., Harrop, K., Drachman, J.G., and Whiting, N. (2010). A phase I multidose study of dacetuzumab (SGN-40; humanized anti-CD40 monoclonal antibody) in patients with multiple myeloma. Haematologica *95*, 845–848.

Johnson, P., Challis, R., Chowdhury, F., Gao, Y., Harvey, M., Geldart, T., Kerr, P., Chan, C., Smith, A., Steven, N., et al. (2015). Clinical and biological effects of an agonist anti-CD40 antibody: a Cancer Research UK phase I study. Clin. Cancer Res. *21*, 1321–1328.

Kabsch, W. (2010). Xds. Acta Crystallogr. D Biol. Crystallogr. *66*, 125–132.

Krissinel, E., and Henrick, K. (2007). Inference of macromolecular assemblies from crystalline state. J. Mol. Biol. *372*, 774–797.

Li, F., and Ravetch, J.V. (2011). Inhibitory Fcgamma receptor engagement drives adjuvant and anti-tumor activities of agonistic CD40 antibodies. Science *333*, 1030–1034.

Li, F., and Ravetch, J.V. (2012). Apoptotic and antitumor activity of death receptor antibodies require inhibitory Fcgamma receptor engagement. Proc. Natl. Acad. Sci. USA *109*, 10966–10971.

Luhder, F., Huang, Y., Dennehy, K.M., Guntermann, C., Muller, I., Winkler, E., Kerkau, T., Ikemizu, S., Davis, S.J., Hanke, T., and Hunig, T. (2003). Topological requirements and signaling properties of T cell-activating, anti-CD28 antibody superagonists. J. Exp. Med. *197*, 955–966.

Martinez, T., Guo, A., Allen, M.J., Han, M., Pace, D., Jones, J., Gillespie, R., Ketchem, R.R., Zhang, Y., and Balland, A. (2008). Disulfide connectivity of human immunoglobulin G2 structural isoforms. Biochemistry *47*, 7496–7508.

McCoy, A.J., Grosse-Kunstleve, R.W., Adams, P.D., Winn, M.D., Storoni, L.C., and Read, R.J. (2007). Phaser crystallographic software. J. Appl. Crystallogr. *40*, 658–674.

Murshudov, G.N., Vagin, A.A., and Dodson, E.J. (1997). Refinement of macromolecular structures by the maximum-likelihood method. Acta Crystallogr. D Biol. Crystallogr. *53*, 240–255.

Pound, J.D., Challa, A., Holder, M.J., Armitage, R.J., Dower, S.K., Fanslow, W.C., Kikutani, H., Paulie, S., Gregory, C.D., and Gordon, J. (1999). Minimal cross-linking and epitope requirements for CD40-dependent suppression of apoptosis contrast with those for promotion of the cell cycle and homotypic adhesions in human B cells. Int. Immunol. *11*, 11–20.

Remer, M., White, A., Glennie, M., Al-Shamkhani, A., and Johnson, P. (2017). The use of anti-CD40 mAb in cancer. Curr. Top. Microbiol. Immunol. *405*, 165–207.

Richman, L.P., and Vonderheide, R.H. (2014). Role of crosslinking for agonistic CD40 monoclonal antibodies as immune therapy of cancer. Cancer Immunol. Res. *2*, 19–26.

Schaer, D.A., Hirschhorn-Cymerman, D., and Wolchok, J.D. (2014). Targeting tumor-necrosis factor receptor pathways for tumor immunotherapy. J. Immunother. Cancer *2*, 7.

Sharma, P., and Allison, J.P. (2015). Immune checkpoint targeting in cancer therapy: toward combination strategies with curative potential. Cell *161*, 205–214.

Smulski, C.R., Beyrath, J., Decossas, M., Chekkat, N., Wolff, P., Estieu-Gionnet, K., Guichard, G., Speiser, D., Schneider, P., and Fournel, S. (2013). Cysteine-rich domain 1 of CD40 mediates receptor self-assembly. J. Biol. Chem. *288*, 10914–10922.

Tao, M.H., and Morrison, S.L. (1989). Studies of aglycosylated chimeric mouse-human IgG. Role of carbohydrate in the structure and effector functions mediated by the human IgG constant region. J. Immunol. *143*, 2595–2601.

Vonderheide, R.H., and Glennie, M.J. (2013). Agonistic CD40 antibodies and cancer therapy. Clin. Cancer Res. *19*, 1035–1043.

Vonderheide, R.H., Flaherty, K.T., Khalil, M., Stumacher, M.S., Bajor, D.L., Hutnick, N.A., Sullivan, P., Mahany, J.J., Gallagher, M., Kramer, A., et al. (2007). Clinical activity and immune modulation in cancer patients treated with CP-870,893, a novel CD40 agonist monoclonal antibody. J. Clin. Oncol. *25*, 876–883.

Werneburg, B.G., Zoog, S.J., Dang, T.T., Kehry, M.R., and Crute, J.J. (2001). Molecular characterization of CD40 signaling intermediates. J. Biol. Chem. *276*, 43334–43342.

White, A.L., Chan, H.T., Roghanian, A., French, R.R., Mockridge, C.I., Tutt, A.L., Dixon, S.V., Ajona, D., Verbeek, J.S., Al-Shamkhani, A., et al. (2011). Interaction with FcgammaRIIB is critical for the agonistic activity of anti-CD40 monoclonal antibody. J. Immunol. *187*, 1754–1763.

White, A.L., Chan, H.T., French, R.R., Beers, S.A., Cragg, M.S., Johnson, P.W., and Glennie, M.J. (2013). FcgammaRIotaIotaB controls the potency of agonistic anti-TNFR mAbs. Cancer Immunol. Immunother. *62*, 941–948.

White, A.L., Dou, L., Chan, H.T., Field, V.L., Mockridge, C.I., Moss, K., Williams, E.L., Booth, S.G., French, R.R., Potter, E.A., et al. (2014).

Fcgamma receptor dependency of agonistic CD40 antibody in lymphoma therapy can be overcome through antibody multimerization. J. Immunol. *193*, 1828–1835.

White, A.L., Chan, H.T., French, R.R., Willoughby, J., Mockridge, C.I., Roghanian, A., Penfold, C.A., Booth, S.G., Dodhy, A., Polak, M.E., et al. (2015). Conformation of the human immunoglobulin G2 hinge imparts superagonistic properties to immunostimulatory anticancer antibodies. Cancer Cell *27*, 138–148.

Wilson, N.S., Yang, B., Yang, A., Loeser, S., Marsters, S., Lawrence, D., Li, Y., Pitti, R., Totpal, K., Yee, S., et al. (2011). An Fcgamma receptor-dependent mechanism drives antibody-mediated target-receptor signaling in cancer cells. Cancer Cell *19*, 101–113.

Winn, M.D., Ballard, C.C., Cowtan, K.D., Dodson, E.J., Emsley, P., Evans, P.R., Keegan, R.M., Krissinel, E.B., Leslie, A.G., McCoy, A., et al. (2011). Overview of the CCP4 suite and current developments. Acta Crystallogr. D Biol. Crystallogr. *67*, 235–242.

Wypych, J., Li, M., Guo, A., Zhang, Z., Martinez, T., Allen, M.J., Fodor, S., Kelner, D.N., Flynn, G.C., Liu, Y.D., et al. (2008). Human IgG2 antibodies display disulfide-mediated structural isoforms. J. Biol. Chem. *283*, 16194–16205.

Xu, Y., Szalai, A.J., Zhou, T., Zinn, K.R., Chaudhuri, T.R., Li, X., Koopman, W.J., and Kimberly, R.P. (2003). Fc gamma Rs modulate cytotoxicity of anti-Fas antibodies: implications for agonistic antibody-based therapeutics. J. Immunol. *171*, 562–568.

Yamniuk, A.P., Suri, A., Krystek, S.R., Tamura, J., Ramamurthy, V., Kuhn, R., Carroll, K., Fleener, C., Ryseck, R., Cheng, L., et al. (2016). Functional antagonism of human CD40 achieved by targeting a unique species-specific epitope. J. Mol. Biol. *428*, 2860–2879.

STAR★METHODS

KEY RESOURCES TABLE

REAGENT or RESOURCE	SOURCE	IDENTIFIER
Antibodies		
Mouse IgG HRP	Dako	Cat. # PO447
Mouse CD23 PE (B3B4)	BD	Cat.# 553139; RRID:AB_394654
Mouse MHCII FITC (M5/114)	In-house	N/A
Mouse CD86 FITC (GL1)	In-house	N/A
Mouse CD8α APC (53-6.7)	BD	Cat. # 561093; RRID: AB_398527
Mouse IgG (FITC)	Abcam	Cat. # ab97264; RRID: AB_ 10688260
Penta.His	Qiagen	Cat. # 34660; RRID: AB_2619735
Human Fc HRP	Jackson ImmunoResearch	Cat. # 109-035-098; RRID: AB_2337586
Human CD23 FITC (MHM6)	In-house	N/A
Human CD40 (Lob 7/4; various isotypes; FITC)	In-house (Johnson et al., 2015)	N/A
Human CD40 (Lob 7/2; various isotypes)	In-house	N/A
Human CD40 (Lob 7/6; various isotypes; FITC)	In-house	N/A
Human CD40 (Lob 7/7; various isotypes; FITC)	In-house	N/A
Human CD40 (Lob 7/8; various isotypes; FITC)	In-house	N/A
Human CD40 (Lob 8/2; various isotypes)	In-house	N/A
Human CD40 (24.2.1; various isotypes)	In-house	N/A
Human CD40 (CP870,893; various isotypes)	In-house (Vonderheide et al., 2007)	N/A
Human CD40 (SGN40; various isotypes)	In-house (Hussein et al., 2010)	N/A
Chemicals, Peptides, and Recombinant Proteins		
Chicken Ovalbumin	Sigma-Aldrich	Cat. # A2512
SIINFEKL tetramer PE	In-house (White et al., 2011)	N/A
Lymphoprep	Axis-Shield	Cat. # 07861
SuperSignal West Pico Chemiluminescent Substrate	Thermo Fisher	Cat. # 34080
GenePORTER	Genlantis	T201075
Critical Commercial Assays		
EasySep™ mouse B cell isolation kit	StemCell Technologies	Cat. # 19854
EasySep™ human B cell isolation kit	StemCell Technologies	Cat. # 17954
Deposited Data		
CD40 ECD/ChiLob 7/4 Fab' crystal structure	Protein Data Bank	6FAX
Experimental Models: Cell Lines		
Freestyle™ 293-F cells	Thermo Fisher Scientific	Cat. # R79007
CHO-K1 cells	Sigma-Aldrich	Cat. # 85051005
NF-κB/Jurkat/GFP reporter cell line	System Biosciences	Cat. # TR850A-1
Experimental Models: Organisms/Strains		
Mouse: C57BL/6	Charles River Laboratories	Strain: 027
Mouse: hCD40Tg: C57BL/6-Tg(hCD40)	Gift from R Noelle (Ahonen et al., 2002)	N/A
Mouse: FcγRII$^{-/-}$: C57BL/6-*Fcgr2*$^{-/-}$	Gift from Dr Verbeek, Leiden University Medical Centre	N/A
Mouse: FcRγ chain $^{-/-}$: C57BL/6-*Fcer1g*$^{-/-}$	Gift from Dr Verbeek, Leiden University Medical Centre	N/A

(*Continued on next page*)

Continued		
REAGENT or RESOURCE	SOURCE	IDENTIFIER
Oligonucleotides		
HuCD40F: AAGCTTGGTCTCACC TCGCCATGGTTCGT		
HuCD40Ec-6HisR: CTCGAGCTAATGATGATGATGATG ATGTCTCAGCCGATGGTG		
Recombinant DNA		
pCIpuro	Modified in-house from PCIneo (Promega Cat. # E1841)	N/A
pEE6.4	Licensed from Lonza	N/A
pEE12.4	Licensed from Lonza	N/A
Software and Algorithms		
Prism	Graphpad	N/A
FCS Express V3	De Novo	N/A
AIMLESS	CCP4 (Evans and Murshudov, 2013)	N/A
PHASER	CCP4 (McCoy et al., 2007)	N/A
COOT	CPP4 (Emsley et al., 2010)	N/A
REFMAC5	CCP4 (Murshudov et al., 1997)	N/A

CONTACT FOR REAGENT AND RESOURCE SHARING

Further information and requests for resources and reagents should be directed to and will be fulfilled by the Lead Contact, Ann L White (ann.white@UCB.com).

EXPERIMENTAL MODEL AND SUBJECT DETAILS

Mice

C57BL/6 mice were from Charles River Laboratories (Kent, UK). *Fcgr2b*$^{-/-}$ and *Fcer1g*$^{-/-}$C57BL/6 mice (Boross et al., 2011) were kindly provided by Dr Sjef Verbeek (Leiden University Medical School). OTI TCR transgenic mice were kindly provided by Dr Matthias Merkenschlager, (Imperial College, London) and human CD40 transgenic (hCD40 Tg) were kindly provided by Randolph Noelle (Kings College, London) (Ahonen et al., 2002). hCD40Tg;*Fcgr2b*$^{-/-}$ and hCD40Tg;*Fcer1g*$^{-/-}$ mice were generated by crossbreeding with genotypes confirmed by flow cytometry. Animals were bred and housed in local animal facilities, were fed regular chow and had freely accessible water. For all experiments, age- (predominantly 8-12 weeks) and sex-matched mice were used that were randomly assigned to experimental groups and housed together under the same conditions. All experiments were carried out according to local ethical committee guidelines under UK Home Office licence numbers PPL30/2451 and PPL30/2964.

Human Samples

PBMCs were obtained from healthy adult volunteers through Southampton National Blood Service after informed consent. As anonymized samples were used, the sex and age of the volunteers are not known. Density gradient centrifugation (Lymphoprep, Axis-Shield) was performed within 4 hours. Use of human samples was approved by the East of Scotland Research Ethics Service, in accordance with the declaration of Helsinki.

METHOD DETAILS

Antibodies and Reagents

Anti-mouse CD23-PE was from BD Biosciences. For OTI cell staining, anti-mouse CD8α−APC (clone 53-6.7; BD Biosciences), and PE- SIINFEKL tetramers were produced in-house as described previously (White et al., 2011). Chicken ovalbumin (OVA) was from Sigma-Aldridge (Poole, UK). Anti-mouse CD86-FITC (clone GL1) and anti-mouse MHCII-FITC (clone M5/114) were both produced in-house.

DNA constructs encoding heavy and light (kappa) chain variable regions of various mAb were either amplified from hybridomas by PCR or synthesized by Genewiz, Inc. The anti-hCD40 mAb SGN40, CP870,893 and 24.2.1 were produced using patent published sequences. Lob 7/2, Lob 7/4, Lob 7/6, Lob 7/7, Lob 7/8 and Lob 8/2 were produced in-house using standard hybridoma technology. Variable regions were subcloned into expression vectors (pEE6.4 for heavy chain and pEE12.4 vector for light chain, Lonza) containing constant regions of different antibody isotypes. Heavy and light chain vectors were subcloned together before transfection into

293F cells (for transient) or CHO-K1 cells (for stable) production of mAb. Secreted mAb were purified by Protein A-Sepharose (Sigma-Aldrich) chromatography and aggregates (as revealed by SEC-HPLC) removed by gel filtration through Sephadex 200 (Sigma-Aldrich). All preparations were endotoxin low (<1 ng/mg protein) as determined by an Endosafe-PTS portable test system (Charles River Laboratories). Non-reducing denaturing capillary electrophoresis (nrCE-SDS) of mAb preparations was performed using a Beckman PA800 Plus analyser according to the manufacturer's instructions. To produce skewed forms of h2, mAb were dialysed into 0.2 M Tris pH8.0 containing 6 mM cysteine plus 1 mM cystamine with (for h2A) or without (for h2B) 2 M guanidine hydrochloride, for 4 days at 4°C, then dialysed into PBS before use.

B Cell Activation and Proliferation

B cells were purified from mouse spleen or human PBMC using magnetic negative selection kits (StemCell Technologies). Cells were plated into 96-well round-bottom dishes at 1 x 10^5 cells/well with concentrations of mAb as described for individual experiments. In some cases, 1 x 10^5 irradiated CHO-K1 or 293F cells transfected with human FcγRIIB (White et al., 2011) were also added. To assess activation, cells were photographed (Olympus CKX41 microscope with CC12 soft imaging system) after 48 hour incubation and activation marker expression analysed by flow cytometry (FACSCalibur, BD Biosciences). Proliferation was assessed by [methyl-^{3}H] thymidine (PerkinElmer, Cambridge, UK) incorporation after 5 days of culture, as described (White et al., 2011).

Immunisation and Assessment of Immune Responses

To assess *in vivo* agonistic activity of anti-hCD40 mAb, 1 x 10^5 splenic OVA-specific CD8 (OT1) T cells were adoptively transferred via tail vein injection in 200 μl PBS; one day later, anti-hCD40 mAb (30 μg or 100 μg, see figure legends) and OVA (100 μg) were injected vial tail vein. Expansion of OT1 cells was monitored by serial blood sampling and of the number circulating enumerated by flow cytometry on a BD FacsCalibur using anti-CD8-APC and SIINFEKL-PE tetramer.

Generation and Expression of CD40 Constructs

A full-length CD40 extracellular domain (ECD) construct (residues 21-193) was generated by PCR using wild-type CD40 DNA as template. The genes for the coding regions of truncated forms of CD40 and CD40-hisTag fusion proteins were synthesized from GeneArt and subcloned into pcDNA3.1 vector. FreeStyle 293F cells (Thermo Fisher) were transfected with constructs using a DNA:PEI (polyethylenimine, Polysciences) ratio of 1:3 and supernatant harvested for purification on day 7 using a Ni-NTA column (GE-Healthcare). To assess binding of mAb to recombinant proteins by western blot, protein samples were prepared in SDS loading buffer without DTT and ran on an 18% polyacrylamide gel. The proteins were transferred on nitrocellulose, the membrane blocked before probing with anti-hCD40 or anti-His mAb (Qiagen) at 4°C overnight. The membrane was washed, then incubated with either goat anti-human Fc-HRP (Jackson Immuno Research) or polyclonal goat anti-mouse IgG-HRP (Dako), and washed before adding SuperSignal West Pico chemiluminescence substrate (Thermo Fisher). Signals were analysed using a Fluor-S Multi-imager Bio-Rad system.

Alanine scanning mutagenesis was carried out on the CRD1 and CRD2 domains of hCD40 to map CD40 mAb epitopes. The wild type extracellular and transmembrane domains of hCD40 were cloned into the pClpuro vector (Promega) for cell surface expression. For scanning mutagenesis, sets of two consecutive residues were mutated to alanines. Nucleotide sequences were designed in house and synthesized by Genewiz (Hertfordshire, UK). CHO-K1 clones stably expressing each mutant were generated using GenePORTER Transfection reagent (Amsbio, UK) and 10 μg/ml puromycin (Invivogen, UK) for selection. Two different stable clones varying in the level of CD40 expression were generated for each mutant. For epitope mapping, 10 μg/ml of hCD40 m1 mAb was incubated with the cells on ice for 30 minutes before secondary detection using goat F(ab')2 anti-mouse Fc-FITC. The level of mAb binding was then assessed by flow cytometry (FACSCalibur) and data analysed by FCS Express V3 (De Novo Software).

Protein Crystallography

ChiLob 7/4-h1 was digested with pepsin (10 mg/ml at room temperature). Cleavage of the heavy chain occurs below the cysteine rich sequence of the hinge region. The cleaved product was subjected to size exclusion chromatography (SEC) using two coupled S200 columns (GE Healthcare; 70ml volume in total) to remove the Fc fragment. The resulting F(ab')$_2$ fragments were reduced with DTT to produce Fab' and quenched by Iodoacetamide. The final Fab' product was verified by HPLC and CE-SDS.

The Fab' fragment was incubated with CD40 ECD (CRD 1-4) in PBS at room temperature for 30 minutes at a molar ratio of 1:4 (CD40:Fab'), followed by SEC using an S200 10/300 column (GE Healthcare) using HEPES buffer (150 mM HEPES, 50 mM KCl pH 7.0). Complex formation was confirmed by HPLC. The complex was concentrated to 13.6 mg/ml for crystallisation in sitting drop plates, set up using a TOPS (Bulek et al., 2012) screen at 20°C using a Gryphon (Art Robbins Instruments). Crystals grew over 3 weeks in condition A11 (20% w/v PEG8000, 0.2 M Ammonium Sulphate, 0.1 M Sodium Cacodylate, pH 6.0) and were harvested in a cryo-protectant (crystallisation buffer containing 20% glycerol), flash frozen and stored under liquid nitrogen.

Diffraction experiments were carried out at beamline ID23-1 (ESRF, France) at a wavelength of 0.9763 (12.7KeV); images were collected using a Pilatus 6M detector. The final dataset in space group P$3_1$21 was processed using XDS to a resolution of 3Å (Kabsch, 2010)

All data manipulation was carried out using the CCP4 suite (Winn et al., 2011). Data reduction and scaling was performed using AIMLESS (Evans and Murshudov, 2013). Molecular replacement was carried out with PHASER using pdb models 1U6A for the Fab' and 3QD6 for CD40 (McCoy et al., 2007). Iterative model building and reciprocal space refinement were carried out using

COOT (Emsley et al., 2010) and REFMAC5 (Murshudov et al., 1997). Refinement statistics are given in Table S1. The final model and structure factors have been deposited in the PDB.

NF-κB Assay

DNA encoding wild-type human CD40 in the pCIpuro vector was transfected into NF-κB/Jurkat/GFP reporter cells (System Biosciences) with Lipofectamine2000 (Thermofisher). Stable transfectants were selected in 10% RPMI medium containing 1 μg/ml puromycin and single-cell cloned to establish a stable cell-line for use. To assess NF-κB activity, cells were stimulated with of anti-hCD40 mAb for 8 hours at 37°C and activity was measured by the production of GFP by flow cytometry.

Surface Plasmon Resonance

A Biacore T100 was used to determine competition between pairs of anti-hCD40 mAb for binding to human CD40. hCD40-Fc (R&D Systems) was immobilised onto a CM5 chip at 1000 RU at pH5. mAb binding was analysed at 500 nM at 25°C with a flow rate of 10 μl/min in HBS-EP+ running buffer (GE Healthcare). The first mAb was injected for 600 seconds followed by the second for 180 seconds. The chip was regenerated with 10 mM glycine pH1.5 for 30 sec at 30 μl/min. The blank control curve was automatically subtracted. Results were analysed using Biacore T100 Evaluation software and GraphPad Prism. mAb used were ChiLob 7/4 h1, SGN40 h1, CP870,893 h1, Lob 7/6 h2.

Phagocytosis Assay

Bone marrow from the femurs and tibia of WT, FcγRIIB KO or γ chain KO C57BL/6 mice was resuspended at $1x10^6$ cells/ml in complete RPMI containing 20% L929 cell supernatant and 50μM β-mercaptoethanol, and incubated for 6-8 days at 37°C/5% CO_2. Adherent macrophages were harvested using trypsin/EDTA (Invitrogen), resuspended in complete RPMI, plated into a 96-well tissue culture plate (5×10^4/well), and incubated for 2 to 4 hours at 37°C/5% CO_2. hCD40Tg B cells were purified as above and labelled with 5 μM CFSE before opsonising with 10 μg/ml of the indicated mAb. Opsonised B cells were then co-cultured with macrophages at a ratio of 5:1 for 30 minutes at 37°C, stained with anti-F4/80-APC and analysed by flow cytometry.

QUANTIFICATION AND STATISTICAL ANALYSIS

Students T-tests (unpaired, two-tailed) or one way ANOVA followed by Tukey's multiple comparisons test were performed using GraphPad Prism software (GraphPad Software, inc., La Jolla, California) as indicated for individual experiments. In some cases data from multiple experiments were combined.

DATA AND SOFTWARE AVAILABILITY

The CD40 ECD/ChiLob 7/4 Fab' crystal structure has been deposited in the PDB database, dataset ID: D_1200001230; PDB ID 6FAX

Cancer Cell, Volume 33

Supplemental Information

Complex Interplay between Epitope Specificity and Isotype Dictates the Biological Activity of Anti-human CD40 Antibodies

Xiaojie Yu, H.T. Claude Chan, Christian M. Orr, Osman Dadas, Steven G. Booth, Lekh N. Dahal, Christine A. Penfold, Lyn O'Brien, C. Ian Mockridge, Ruth R. French, Patrick Duriez, Leon R. Douglas, Arwen R. Pearson, Mark S. Cragg, Ivo Tews, Martin J. Glennie, and Ann L. White

Table S1. Related to Figure 2.

	ChiLob 7/4 Fab:CD40 Complex
**Data collection**	
**Space group**	P 3_1 2 1
**Cell dimensions**	
**a, b, c (Å)**	158.75 158.75 96.62
**a, b, g (°)**	90 90 120
**Resolution (Å)**	51.96 - 3.00 (3.08-3.00)
**R_{sym} or R_{merge}**	0.157 (1.445)
**I / sI**	13.0 (32.9)
**Completeness (%)**	100 (98.4)
**Redundancy**	13.7 (11.4)
**Refinement**	
**Resolution (Å)**	3.00
**No. reflections**	27540
**R_{work} / R_{free}**	0.190 / 0.237
**No. atoms**	
**Protein**	4091
**Ligand/ion**	0
**Water**	23
**B-factors**	
**Protein**	87.03
**Ligand/ion**	0
**Water**	78.14
**R.m.s. deviations**	
**Bond lengths (Å)**	0.0169
**Bond angles (°)**	2.135

Data Collection and Refinement Statistics of ChiLob 7/4 Fab':CD40 Complex (PDB ID 6FAX). High resolution shell statistics shown in parenthesis. 5% of reflections were used for calculation of Rfree.

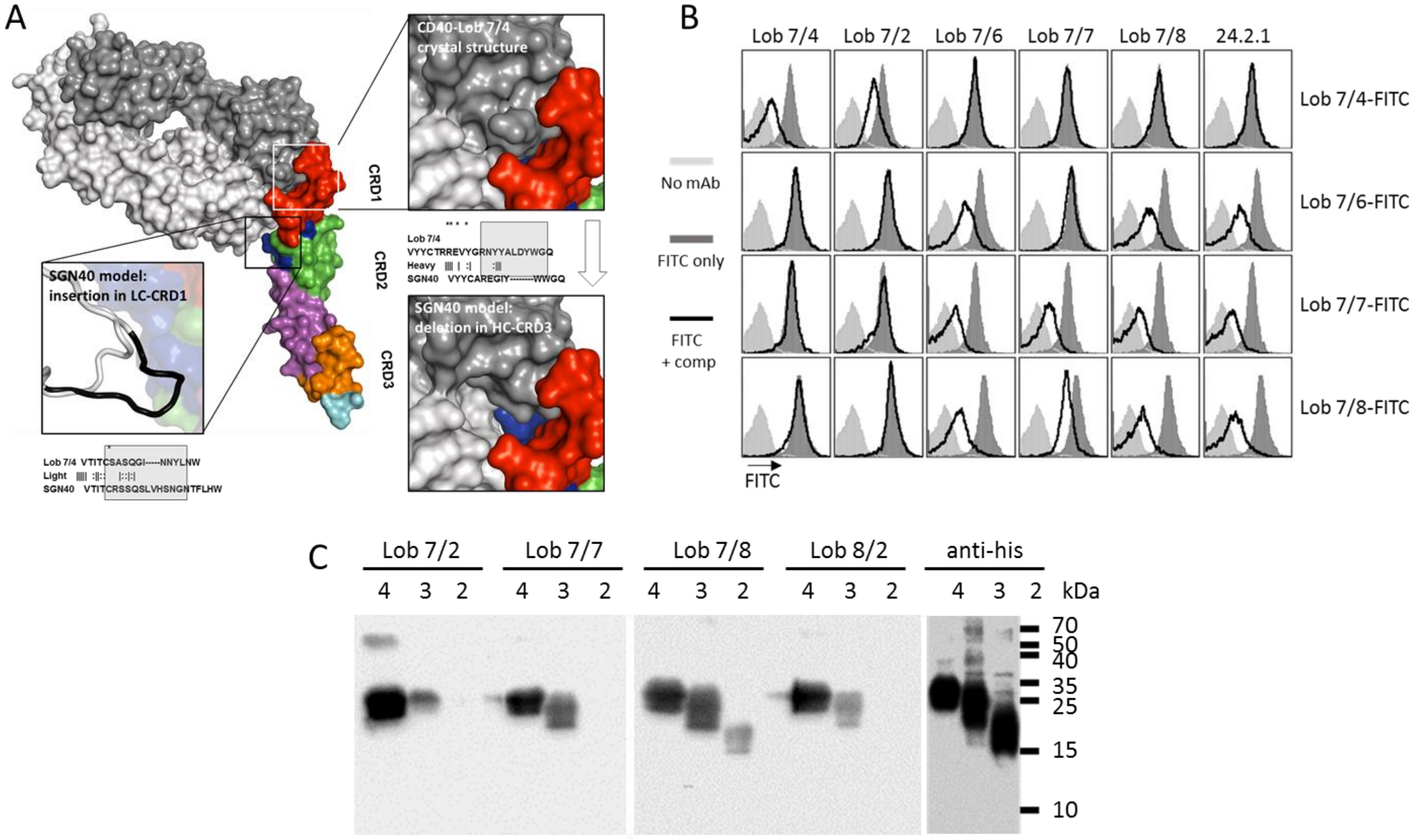

Figure S1. Epitope mapping of hCD40 mAb. Related to Figure 3. (A) Modelling of SGN40 binding onto the ChiLob 7/4:CD40 crystal structure. Surface view of the structure of the CD40:ChiLob 7/4 complex with CRD regions of CD40 coloured and mapped as in Figure 2 with various insets highlighting key parts of the interaction. Below are models produced to show the predicted structural differences apparent in the SGN40 and ChiLob 7/4 Fab' based on loop length changes in the heavy chain CRD3 and the light chain CRD1. Panels: Top right panel highlights the binding of ChiLob 7/4 to CD40 in the crystal structure. Note: The LC CDR3 of ChiLob 7/4 binds into a pocket on the CRD1-B2 of CD40 and the light chain CRD1 of ChiLob 7/4 binds in a pocket of CD40 CDR1-A1. Below is shown the sequence alignment between the HC CDR3 of ChiLob 7/4 and SGN40 with the key binding residues in the structure of ChiLob 7/4 starred. Below (bottom right) shows how the 8 amino-acid deletion in the SGN40 HC would prevent it making the same interaction with the pocket of CRD1-B2 of

CD40 as ChiLob 7/4. The bottom left panel shows a modelled representation of how the 5 amino-acid insertion of the SGN40 LC CDR1 (bottom loop, black) would impair binding in this region. Below is the sequence alignment between the LC CDR1 of ChiLob 7/4 and SGN40 with the key binding residue starred. (B) hCD40Tg mouse B cells were incubated with FITC-labelled Lob 7/4, Lob 7/6, Lob 7/7 or Lob 7/8 as indicated in the absence or presence of a 10-fold excess of competitor (comp) mAb indicated above each plot. Cell labelling was assessed by flow cytometry; light grey, unlabelled cells; dark grey, FITC-labelled mAb alone; black line, FITC-labelled mAb + competitor. (C) C-terminally His-tagged CD40 proteins consisting of 4, 3, or 2 CRD domains (4 = full length; 3 = CRD1 deleted; 2 = CRD1 and 2 deleted) were analysed by Western blotting with the mAb indicated above each plot used for detection. A composite image from multiple blots is shown.

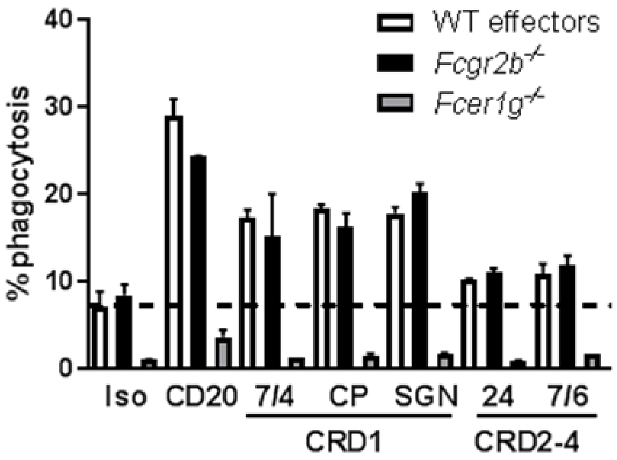

Figure S2. anti-hCD40 mAb-mediated phagocytosis. Related to Figure 4. Purified hCD40Tg mouse splenic B cells were opsonised with the indicated hCD40 mAb, mouse CD20 mAb, or isotype control, all m2a isotype, before incubation with bone marrow derived mouse macrophages from WT, *Fcgr2b*$^{-/-}$ or *Fcer1g*$^{-/-}$ mice. Phagocytosis was measured by flow cytometry as described in STAR Methods. Results (mean ± SEM) from one of 2 independent experiments are shown, * $p<0.05$ versus isotype control for WT cells.

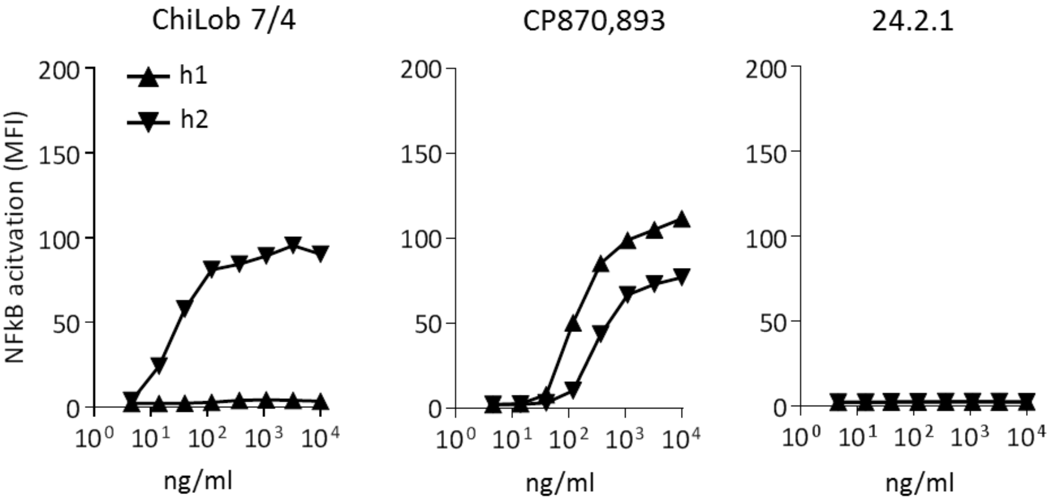

Figure S3. NF- κ B activation by anti-hCD40 mAb. Related to Figure 5. NF-κB/Jurkat/GFP reporter cells stably transfected with hCD40 were incubated with increasing concentrations of the indicated anti-hCD40 mAb of h1 or h2 isotypes for 8 hours, and then the level of NF- κ B activation was assessed by GFP quantification using flow cytometry. Mean ± SEM of triplicates, data representative of at least 2 independent experiments.
